# Carbon dot- and nanoencapsulated essential oil-enabled electrospun microfibers mitigate oxidative deterioration and extend the shelf life of fresh-cut pomegranate arils

**DOI:** 10.1016/j.fochx.2026.104441

**Published:** 2026-09-22

**Authors:** Sahar Ashari, Raheleh Ebrahimi, Weria Weisany, Shima Yousefi, Orang Khademi

**Affiliations:** a Department of Horticultural Science, Agronomy and Biotechnology, SR.C., Islamic Azad University, Tehran, Iran; b Department of Food Science, SR.C., Islamic Azad University, Tehran, Iran; c Department of Horticulture, Faculty of Agriculture, Shahed University, Tehran, Iran

**Keywords:** Active packaging, Antimicrobial activity, Carbon dots, Electrospun microfibers, Nanoencapsulated essential oil, Shelf-life

## Abstract

Fresh-cut pomegranate arils rapidly deteriorate due to oxidative stress, microbial growth, membrane damage, and loss of bioactive compounds. This study developed a multifunctional electrospun poly(vinyl alcohol)/chitosan (PVA/CH) microfibrous pad incorporating carbon dots (CDs), Persian hogweed essential oil (EO), and nanoencapsulated EO (EONCs) to improve postharvest preservation. Four treatments (control, CDs, EO, and EONCs) were evaluated during 20 days of refrigerated storage. Structural characterization confirmed that CDs possessed functionalities, fluorescence, and an amorphous carbon structure, while EONCs exhibited high encapsulation efficiency for sustained release. Compared with controls, nano-enabled packaging reduced weight loss, electrolyte leakage, and microbial growth while preserving acidity, soluble solids, sugars, phenolics, antioxidant activity, and ascorbic acid. Activities of CAT, SOD, POD, PPO, and PAL were moderated, indicating reduced reactive oxygen species accumulation and delayed senescence. The combined CD/EONC system provides a synergistic strategy for enhancing physicochemical stability, maintaining fruit quality, and extending shelf life of fresh-cut pomegranate arils.

## Highlights

• Electrospun PVA/chitosan pads loaded with CDs and EO nanocarriers were developed.
• Nano-enabled pads suppressed microbial growth in fresh-cut pomegranate arils.
• Carbon dots and EO nanocarriers reduced oxidative deterioration during storage.
• Active nanofibers preserved phenolics, ascorbic acid, and antioxidant capacity.
• Synergistic redox regulation delayed senescence and extended aril shelf life.

## 1 Introduction

Fresh and minimally processed fruits are increasingly demanded because of their convenience, nutritional value, and potential health benefits. Among these products, pomegranate (*Punica granatum* L.) is recognized as a “superfruit” because of its phenolic compounds, anthocyanins, organic acids, vitamins, and antioxidant capacity (Nizama-Chapoñan et al., 2020). The ‘Saveh Sweet’ cultivar also reported as ‘Sweet Saveh’ in English-language literature is among the commercially harvested Iranian pomegranate cultivars and is associated with Saveh, one of Iran's principal pomegranate-producing regions (Mohammadi and Mirghazanfari, 2019). It is classified as a sweet cultivar suitable for fresh consumption, while studies of Iranian Saveh cultivars have demonstrated cultivar-dependent profiles of anthocyanins, phenolic compounds, and antioxidant activity (Mousavinejad et al., 2009). These characteristics support its commercial relevance for fresh and minimally processed products. However, separating the arils removes the protective peel and internal membranes, disrupts cellular compartmentalization, and increases moisture loss, respiration, oxidative reactions, and susceptibility to microbial contamination, resulting in rapid deterioration during refrigerated storage (Zaman et al., 2025).

Pomegranate is an economically important fruit, with global production estimated at approximately 3.8 million tonnes and Iran contributing about 1.34 million tonnes annually. Ready-to-eat arils offer commercial value by overcoming the inconvenience of peeling and meeting consumer demand for convenient products (Valero-Mendoza et al., 2023). However, fresh-cut processing removes the fruit's natural protective barriers, accelerating moisture loss, microbial growth, and quality deterioration, thereby creating a need for effective active-packaging systems.

Postharvest deterioration of fresh-cut pomegranate arils is governed by interconnected physiological and biochemical processes. Oxidative stress plays a central role in senescence progression. During storage, reactive oxygen species (ROS), including superoxide radicals (O₂^−^•) and hydrogen peroxide (H₂O₂), accumulate as byproducts of metabolic activity (Meitha et al., 2020). Although plant tissues possess endogenous antioxidant enzymes, including superoxide dismutase (SOD), catalase (CAT), and peroxidase (POD), together with stress-related enzymes such as polyphenol oxidase (PPO) and phenylalanine ammonia-lyase (PAL), excessive production of reactive oxygen species can overwhelm these protective responses (Hajam et al., 2023). The result is lipid peroxidation, membrane destabilization, electrolyte leakage, phenolic oxidation, enzymatic browning, and depletion of ascorbic acid.

Simultaneously, microbial proliferation significantly accelerates postharvest degradation (Weisany, Samadi, et al., 2022). Mesophilic bacteria, yeasts, and molds utilize soluble sugars and organic acids as metabolic substrates, increasing respiration rates and generating additional oxidative stress. Microbial metabolism also produces extracellular enzymes and oxidative byproducts that compromise tissue integrity (Almalki, 2021). Therefore, oxidative imbalance and microbial growth function synergistically, creating a deterioration cascade that affects physicochemical stability, nutritional retention, and sensory quality (Martínez & Carballo, 2021; Ogwu & Ogunsola, 2024).

Conventional preservation approaches, including low-temperature storage and modified atmosphere packaging, partially slow deterioration but do not adequately control oxidative stress and microbial proliferation simultaneously. In recent years, active packaging technologies incorporating nanomaterials have emerged as promising multifunctional strategies for fresh produce preservation (Pattnaik et al., 2024; Wang et al., 2025). Unlike passive packaging, active systems are designed to interact with the packaged food environment, modulating gas composition, humidity, microbial load, and oxidative reactions (Du et al., 2023; Kadirvel et al., 2025).

Carbon dots, a class of zero-dimensional carbon-based nanomaterials, have attracted significant attention due to their unique physicochemical and optical properties (Anagnostou et al., 2023). CDs typically possess abundant surface functional groups, including hydroxyl, carbonyl, and carboxyl moieties, which enhance water dispersibility and enable redox-active behavior (Mansuriya and Altintas, 2021b). Their partially graphitized yet predominantly amorphous structure provides surface defect states responsible for photoluminescence and electron transfer capabilities (Yao et al., 2022). Recent studies suggest that CDs can act as reactive oxygen species modulators, electron donors/acceptors, and antimicrobial agents due to their high surface area and surface charge characteristics (Romulo et al., 2025; Sheikh Mohd Ghazali et al., 2023). These properties position CDs as potential nano-enabled stabilizers capable of mitigating oxidative stress and limiting microbial proliferation in postharvest systems.

Parallel to nanomaterial-based preservation strategies, plant-derived essential oils have attracted interest as natural antimicrobial and antioxidant agents. *Heracleum persicum* Desf. ex Fisch. (Apiaceae), commonly known as Persian hogweed or Golpar, is an aromatic plant native to Iran and traditionally used as a culinary spice and in folk medicine. In the present study, EO was hydrodistilled specifically from dried aerial shoots collected at flowering. GC–MS analysis indicated an ester-rich volatile profile dominated by hexyl butanoate and *n*-octyl acetate, with ethylidenecyclohexane and octyl butanoate as other abundant constituents and smaller amounts of *p*-cymene, γ-terpinene, and linalool. Compounds identified solely through mass-spectral-library and retention-index matching were considered tentative. The hydrophobic constituents of EOs may interact with microbial cell envelopes and contribute to antimicrobial activity; however, their effectiveness depends strongly on composition and delivered concentration (Bansal et al., 2023). Direct application of free EO is limited by volatility, poor aqueous dispersibility, rapid depletion, and intense aroma, potentially reducing sustained activity and adversely affecting sensory quality (Al-Maqtari et al., 2022; Weisany, Yousefi, & Tahir, N.A.-r., Golestanehzadeh, N., McClements, D.J., Adhikari, B., Ghasemlou, M., 2022).

Nanoencapsulation offers a solution to these drawbacks. Encapsulating EO within biopolymeric matrices such as chitosan enhances stability, reduces premature evaporation, improves dispersibility, and enables controlled release of bioactive compounds (Detsi et al., 2020). Chitosan, a biodegradable and biocompatible polysaccharide, also exhibits intrinsic antimicrobial activity due to its positively charged amino groups, which interact with negatively charged microbial membranes (Ke et al., 2021). Therefore, combining chitosan-based nanoencapsulation with ester-rich EO may generate a synergistic antimicrobial system with prolonged activity.

Electrospinning technology offers a versatile and highly efficient platform for incorporating nanomaterials and encapsulated bioactive compounds into advanced packaging systems (Munteanu & Vasile, 2021). Owing to their extremely high surface area-to-volume ratio, interconnected porous structure, and tunable fiber morphology, electrospun microfibers provide enhanced loading capacity and controlled release behavior compared with conventional films. Blending PV) with CH results in stable, biocompatible microfibrous mats exhibiting suitable mechanical strength, moisture-regulating properties, and intrinsic antimicrobial potential (Chatterjee et al., 2026). The incorporation of redox-active CDs and nanoencapsulated EO into PVA/CH electrospun pads enables the development of a multifunctional active packaging system. Such a system can simultaneously modulate oxidative stress through redox-mediated interactions, suppress microbial proliferation via sustained release of bioactive compounds, preserve cellular membrane integrity by reducing electrolyte leakage, stabilize key metabolites including sugars, organic acids, phenolics, and ascorbic acid, and maintain physicochemical and color attributes during refrigerated storage. Collectively, this integrated nanocomposite strategy provides a synergistic mechanism for enhancing postharvest quality and extending the shelf life of fresh produce.

Most previously reported electrospun active-packaging systems have relied on a single active component or the direct incorporation of free EOs. Although direct EO loading can provide antimicrobial and antioxidant activity, its effectiveness is frequently limited by poor aqueous compatibility, volatility, rapid diffusion, and an initial burst followed by premature depletion. Carbon-dot-containing packaging systems, in contrast, have generally focused on their surface functionality, optical properties, or individual antimicrobial and antioxidant potential, with limited attention to their integration with controlled-release EO nanocarriers. The present system differs by co-incorporating water-dispersible carbon dots and nanoencapsulated Persian hogweed (*H. persicum*) EO within a polyvinyl alcohol/chitosan electrospun matrix. Nanoencapsulation was intended to protect the volatile EO constituents and prolong their availability, whereas the carbon dots provided an additional functional component distributed throughout the hydrophilic microfibrous network. Their integration was therefore expected to provide complementary protection against oxidative deterioration and microbial proliferation across different stages of storage. Importantly, this proposed complementarity was treated as a hypothesis rather than evidence of experimentally confirmed synergy.

Accordingly, this study aimed to: (i) characterize the surface chemistry, optical behavior, structural organization, and hydrodynamic particle-size distribution of the carbon dots using Fourier-transform infrared spectroscopy, photoluminescence spectroscopy, X-ray diffraction, and dynamic light scattering; (ii) prepare nanoencapsulated Persian hogweed EO and determine its particle size, encapsulation efficiency, physicochemical stability, and release behavior; (iii) fabricate polyvinyl alcohol/chitosan electrospun pads containing carbon dots and nanoencapsulated EO and characterize their morphology and structural interactions; and (iv) determine how the nano-enabled pads and storage duration affected the physicochemical, biochemical, enzymatic, microbial, and nutritional characteristics of fresh-cut pomegranate arils. We hypothesized that co-localizing carbon dots with sustained-release EO nanocarriers would provide more persistent protection than direct incorporation of free EO by reducing its initial burst and maintaining the active compounds throughout refrigerated storage. This combined functionality was expected to limit microbial growth and membrane deterioration and improve the retention of antioxidant and nutritional quality; however, formal synergy would require a factorial interaction analysis and direct antioxidant testing of the individual and combined pads.

## 2 Materials and methods

### 2.1 Plant material and fruit preparation

Mature ‘Saveh Sweet’ pomegranate (*Punica granatum* L.), a commercially important cultivar widely cultivated in Iran, was harvested during the early morning hours in the first week of October from a commercial orchard in Saveh, Markazi Province, Iran. Fruits exhibiting uniform size, color, and weight and free from visible defects, sunburn, mechanical injuries, pest infestation, or disease symptoms were selected for the study. Immediately after harvest, the fruits were transported to the laboratory and equilibrated under refrigerated conditions (4 ± 1 °C, 90 ± 5% relative humidity) for 24 h prior to minimal processing.

### 2.2 Processing and preparation of arils

Prior to minimal processing, fruits were washed with distilled water and aseptically cut along the equatorial plane using a sterile stainless-steel knife. The peel and internal membranous tissues were manually removed, and arils were carefully separated to minimize mechanical damage. The arils were subsequently sanitized by immersion in a sodium hypochlorite solution (NaClO, pH 6.5) at 5 °C for 2 min, followed by rinsing with chilled distilled water (5 °C) for 1 min to remove residual sanitizer. Excess surface moisture was eliminated using a centrifugal spin dryer for 1 min, and the arils were subsequently equilibrated at 8 °C for 1 h to ensure complete removal of residual water. The prepared arils were then randomly allocated into four treatment groups for subsequent packaging applications.

### 2.3 Experimental design and storage conditions

The experiment was arranged as a two-factor factorial study within a completely randomized design (CRD) with three independent replicates. The investigated factors comprised four packaging treatments, including untreated control, CDs, Persian hogweed EO, and nanoencapsulated Persian hogweed EO (EONCs), as well as five storage periods (1, 5, 10, 15, and 20 days). CDs were purchased from Shimi Sanat Roshd Sahand Co. (Tabriz, Iran), an Iranian knowledge-based manufacturer of carbon-based quantum dots. The active compounds were incorporated into electrospun absorbent microfibrous pads, which were positioned at the base of each packaging container to enable sustained release of bioactive constituents during storage. Approximately 200 g of fresh-cut pomegranate arils were packaged in each container and stored at 4 ± 1 °C under refrigerated conditions. Physicochemical, biochemical, microbiological, enzymatic, and nutritional quality attributes were determined at predefined sampling intervals throughout the storage period.

### 2.4 Essential oil extraction

Fresh aerial shoots of *H. persicum*, comprising the aboveground stems, leaves, and flowering tissues, were collected at the flowering stage. The plant material was shade-dried at room temperature and subsequently ground into a fine powder. A 100 g portion of the dried powder was mixed with 1000 mL of distilled water and subjected to hydrodistillation for 3 h using a Clevenger-type apparatus. The recovered EO was separated, dried over anhydrous sodium sulfate to remove residual moisture, filtered, transferred into sealed amber-glass vials, and stored at 4 °C until analysis and application. EO yield was expressed as mL of oil per 100 g of dry plant material (% *v*/*w*). The extraction procedure was performed independently in triplicate.

### 2.5 Preparation of essential oil nanocapsules

Essential oil nanocapsules (EONCs) were prepared using a high-shear homogenization-solvent evaporation approach. Briefly, 40 mL of a chitosan/poly(vinyl alcohol) (PVA) aqueous solution containing 0.5% (*v*/*v*) acetic acid was transferred into a cylindrical ceramic vessel and pre-homogenized for 10 s to ensure complete mixing. The homogenizer probe was positioned approximately 1 cm above the bottom of the vessel to achieve efficient energy dissipation and uniform shear distribution. Subsequently, 5 mL of ethyl acetate containing 30% (*v*/*v*) Persian hogweed EO was added dropwise under continuous homogenization, and emulsification was continued for 20 min to obtain a stable oil-in-water nanoemulsion. The resulting dispersion was transferred into sealed glass vials and magnetically stirred overnight at 500 rpm under ambient conditions to facilitate complete evaporation of the organic solvent and formation of EO-loaded nanocapsules. For purification, 10 mL aliquots of the nanocapsule suspension were centrifuged at 6000 rpm for 45 min, and the resulting pellet was re-dispersed in HPLC-grade water. The suspension was subsequently subjected to ultrasonication followed by centrifugation to remove residual free polymer, non-encapsulated EO, and solvent traces. This purification cycle was repeated three times prior to physicochemical characterization and incorporation into the active packaging system (Li et al., 2026).

### 2.6 Fabrication of electrospun active microfibrous pads

Electrospun microfibrous pads were fabricated using a laboratory-scale electrospinning system under optimized operating conditions. Electrospinning was conducted at a constant feed rate of 1 mL h^−1^, an applied voltage of 20–27 kV, and a tip-to-collector distance of 100 mm. Microfibers were deposited onto a flat collector to obtain uniform mats with a final surface area of approximately 300 cm^2^. The control microfibrous pad was prepared from a PVA/CH polymer blend composed of 10 g PVA and 5 g CH. Approximately 2 mL of the polymer solution was electrospun through a single-nozzle configuration to produce continuous and bead-free microfibrous mats. For the preparation of CD-loaded microfibers, 300 mg of CDs were dispersed in 3 mL dimethyl sulfoxide (DMSO) under magnetic stirring for 10 min. Subsequently, 3 mL of the PVA/CH solution was added and stirred for an additional 15 min to ensure homogeneous distribution of CDs within the polymer matrix. The resulting suspension was electrospun using a single-nozzle system for 4 h, during which approximately 4 mL of solution was deposited onto the collector to obtain CD-incorporated microfibrous pads.

Microfibrous pads containing Persian hogweed EO were fabricated either by direct incorporation of free EO or by incorporating EONCs. For the free EO formulation, 5 mL of EO was emulsified with 1 mL Tween 80 and subsequently mixed with 5 mL of the PVA/CH solution under magnetic stirring for 15 min to obtain a homogeneous electrospinning suspension. For the nanoencapsulated formulation, 4 mL of chitosan-based EONCs was blended with 2 mL of the PVA/CH solution and stirred for an additional 15 min. Both EO-containing formulations were electrospun using a dual-nozzle configuration for 2 h, during which approximately 4 mL of solution was continuously supplied to produce active microfibrous pads containing either free EO or EONCs (Iurascu et al., 2026).

Following electrospinning, the microfibrous mats were dried at 25 ± 2 °C for 24 h and subsequently conditioned in a vacuum desiccator for an additional 24 h to minimize residual moisture and solvent. Drying and conditioning before characterization are commonly applied to EO-loaded electrospun microfibers to stabilize their mass and structure and reduce interference from residual solvent (Moradinezhad et al., 2024). The mats were cut into 5 × 5 cm pads, providing an exposed surface area of 25 cm^2^. The mean thickness and dry mass of each conditioned pad were standardized to 0.20 ± 0.02 mm and 0.250 ± 0.015 g, respectively. Control and active pads were prepared with identical dimensions and comparable dry masses because the amount and exposed surface area of an EO-loaded material can substantially influence volatile release and antimicrobial performance (Casalini et al., 2023).

Based on the formulation and mass balance of the electrospinning solutions, the nominal CD content was standardized to 25 mg pad^−1^, equivalent to 100 mg CDs g^−1^ dry microfiber and 1.0 mg CDs cm^−2^ pad area. The free-EO and EONC pads were formulated to provide an equivalent nominal Persian hogweed EO loading of 25 mg pad^−1^, corresponding to 100 mg EO g^−1^ dry microfiber and 1.0 mg EO cm^−2^. Equal EO loading was used in the free and nanoencapsulated formulations to permit direct comparison of their release kinetics and preservation efficacy. Expressing the active-agent content relative to both the mass and surface area of the carrier is recommended because these parameters affect the concentration of volatile compounds delivered to the package headspace (Casalini et al., 2023; Moradinezhad et al., 2024). The actual retained loading was determined by extracting the EO from a known mass of microfibers followed by gas chromatography analysis and was calculated as the mass of recovered EO per gram of dry pad.

For the storage experiment, 200 ± 1 g of freshly separated pomegranate arils was placed in food-grade polyethylene terephthalate clamshell containers. A 200-g sample size has previously been used in refrigerated-storage experiments involving minimally processed pomegranate arils (El-Beltagi et al., 2023). The containers had internal dimensions of approximately 13.5 × 11.0 × 3.0 cm and a nominal internal volume of approximately 445 mL. Comparable food-grade clamshell packages have been used to evaluate active films releasing EO constituents into the package headspace (Casalini et al., 2023). The volume displaced by the arils was approximately 195 mL; therefore, the effective package headspace was standardized to approximately 250 mL.

One 5 × 5 cm microfibrous pad was attached horizontally to the inner surface of the container lid using food-grade double-sided adhesive, with the active surface facing the arils. The pad was positioned approximately 1.0 cm above the product and did not directly contact the fruit. Positioning the active material on the inner package lid permits vapor-phase transfer of EO constituents while preventing direct contact between the active pad and the food (Casalini et al., 2023). The pad-to-product ratio and exposed pad-area-to-product ratio were 1.25 g kg^−1^ and 125 cm^2^ kg^−1^, respectively. For the EO-containing treatments, the nominal EO-to-headspace ratio was 100 mg L^−1^. Reporting EO loading relative to headspace volume is important because vapor-phase antimicrobial activity may depend more strongly on the EO concentration per L of headspace than on its concentration per unit mass of the carrier material (Casalini et al., 2023).

The containers were closed with their original fitted lids and sealed around the perimeter using a food-grade polyethylene sealing film to minimize uncontrolled loss of volatile EO constituents. Package sealing and headspace conditions influence EO diffusion, accumulation, and antimicrobial effectiveness and were therefore kept identical among treatments (Casalini et al., 2023; Moradinezhad et al., 2024). Identical package geometry, product mass, pad position, headspace volume, and storage conditions were maintained for all treatments. The prepared packages were stored at 4 ± 1 °C and 90 ± 5% relative humidity for 20 days. Comparable temperatures have been used to maintain minimally processed pomegranate arils under refrigerated storage (El-Beltagi et al., 2023).

### 2.7 Fourier transform infrared (FTIR)

The surface chemistry and functional groups of the synthesized nanoparticles were characterized by FTIR spectroscopy. Freeze-dried nanoparticle samples were finely ground and homogenized with spectroscopic-grade potassium bromide (KBr) prior to pellet formation. FTIR spectra were acquired over the wavenumber range of 4000–400 cm^−1^ using an FTIR spectrometer at a spectral resolution of 4 cm^−1^, with 32 scans accumulated for each sample to improve the signal-to-noise ratio (Stuart, 2021). A background spectrum was collected and automatically subtracted before each analysis. The resulting spectra were interpreted to identify characteristic vibrational bands associated with specific chemical functionalities and surface moieties present on the nanoparticles.

### 2.8 Photoluminescence (PL) spectroscopy

The optical properties of the synthesized nanoparticles were investigated by PL spectroscopy. Nanoparticle suspensions were prepared in deionized water and ultrasonicated prior to analysis to ensure homogeneous dispersion and minimize particle aggregation. PL measurements were carried out at room temperature using a fluorescence spectrophotometer. The samples were excited at a selected excitation wavelength (λ_ex), and the corresponding emission spectra were recorded over the designated wavelength range to determine fluorescence emission characteristics, including emission maxima and relative fluorescence intensity. All measurements were performed in quartz cuvettes with a 1 cm optical path length. The acquired spectra were analyzed to assess the optical behavior of the nanoparticles and to verify the presence of emissive surface states associated with their carbonaceous structure.

### 2.9 X-ray diffraction

The crystalline structure and degree of structural ordering of the synthesized nanoparticles were investigated by X-ray diffraction (XRD) analysis. Freeze-dried nanoparticle powders were uniformly spread and gently compacted onto the sample holder to obtain a flat and homogeneous surface. XRD patterns were acquired using an X-ray diffractometer equipped with Cu Kα radiation (λ = 1.5406 Å) operated at 40 kV and 30 mA. Diffraction data were collected over a 2θ range of 5–70° at a scanning rate of 2° min^−1^ (Aly et al., 2024). The resulting diffraction patterns were interpreted to evaluate the degree of crystallinity, structural ordering, and the presence of amorphous and graphitic domains characteristic of carbon-based nanomaterials

### 2.10 Encapsulation efficiency

EE of the EO-loaded nanoparticles was determined by quantifying the amount of non-encapsulated (free) EO present in the supernatant after nanoparticle separation. Briefly, a known volume of freshly prepared nanoparticle suspension was centrifuged at 10,000–15,000 rpm for 30–45 min at 4 °C to separate the nanoparticles from the aqueous phase. The supernatant containing the unencapsulated EO was carefully collected without disturbing the pellet.

The concentration of free EO in the supernatant was determined spectrophotometrically by UV–Vis analysis. Absorbance was measured at the characteristic maximum wavelength (λ_max) of the EO, and concentrations were calculated using a previously constructed standard calibration curve (R^2^ = 0.995). All measurements were performed in triplicate. Based on these measurements, the encapsulation efficiency of the nanoparticles was 87.6 ± 2.4%, indicating efficient entrapment of the EO within the polymeric matrix.

Encapsulation efficiency was calculated according to the following equation:

$$\mathrm{EE}\left(\%\right)=\frac{\left(\mathrm{Total}\;\mathrm{EO}-\mathrm{Free}\;\mathrm{EO}\right)}{\mathrm{Total}\;\mathrm{EO}}\times 100$$

where *Total EO* represents the initial amount of EO used in nanoparticle preparation, and *Free EO* corresponds to the amount detected in the supernatant after centrifugation (Murtadza et al., 2021).

### 2.11 Weight loss

Weight loss was monitored throughout storage to evaluate moisture loss and dehydration behavior of treated samples. At each sampling interval (1, 5, 10, 15, and 20 days), each package was weighed using a high-precision analytical balance (±0.001 g). Initial weight (W₁) was recorded immediately after packaging, and subsequent weights (W₂) were recorded at each sampling day. Weight loss was calculated using the following equation:

$$\text{Weight loss}\;\left(g\right)=W_{1}-W_{2}$$

### 2.12 Color measurement (L*, a*, b*, C*, h°)

Surface color of samples was evaluated using a calibrated chroma meter based on the CIE Lab system. The instrument was standardized using a white calibration plate prior to measurements. For each replicate, three random readings were taken and averaged.

The parameters recorded were:

L* (lightness, 0 = black, 100 = white).

a* (redness/greenness; +a* = red, −a* = green).

b* (yellowness/blueness; +b* = yellow, −b* = blue).

Chroma (color saturation) and hue angle were calculated as:

$$C^{*}=\sqrt{a^{*2}+b^{*2}}$$

$$h^{\circ }=\tan^{-1}\left(b^{*}/a^{*}\right).$$

These parameters allowed evaluation of pigment stability and browning development during storage.

### 2.13 pH

Fresh juice was extracted from 50 g of sample tissue using a sterile manual press and filtered through cheesecloth to remove solid residues. The pH of the filtrate was measured at room temperature using a calibrated digital pH meter. The instrument was standardized prior to use with buffer solutions of pH 4.0 and 7.0 to ensure accuracy. Measurements were performed in triplicate for each treatment.

### 2.14 Titratable acidity

Titratable acidity (TA) was determined according to the method described by Paul et al. (2010) with minor modifications. Briefly, 10 mL of freshly extracted pomegranate juice was diluted with 50 mL of distilled water and titrated against 0.1 M NaOH to an endpoint of pH 8.1 using a digital burette under continuous magnetic stirring. Titratable acidity was calculated and expressed as grams of citric acid equivalent per 100 g fresh weight (g citric acid 100 g^−1^ FW). All determinations were performed in triplicate (Paul et al., 2010).

### 2.15 Total soluble solids

Total soluble solids (TSS) were determined using a digital refractometer at 20 ± 1 °C according to the method of Kimball (1991). Briefly, several drops of filtered pomegranate juice were placed onto the prism surface, and the readings were recorded as °Brix. The refractometer was calibrated with distilled water prior to each measurement session. All analyses were performed in triplicate, and the results were expressed as °Brix (Kimball, 1991).

### 2.16 Firmness

Fruit firmness was evaluated using a texture analyzer fitted with a cylindrical stainless-steel probe. Arils were subjected to a penetration test at a constant crosshead speed, and the maximum force required to penetrate the tissue was recorded as an indicator of firmness. The results were expressed in Newtons (N). To account for sample heterogeneity and minimize experimental variability, measurements were performed at multiple locations within each replicate, and the mean value was used for statistical analysis.

### 2.17 Total sugars

Total sugar content was quantified using the phenol‑sulfuric acid colorimetric method according to Nielsen (2009). Briefly, an aliquot of the sample extract was mixed with 5% (*w*/*v*) phenol solution, followed by the rapid addition of concentrated sulfuric acid. The reaction mixture was incubated at room temperature to allow complete color development, and the absorbance was measured at 490 nm using a UV–Vis spectrophotometer. Glucose solutions of known concentrations were used to generate a calibration curve, and total sugar content was calculated from the corresponding regression equation. The results were expressed as g glucose equivalent per 100 g fresh weight (g GE 100 g^−1^ FW) (Nielsen, 2009). All analyses were performed in triplicate.

### 2.18 Total carbohydrates

Total carbohydrate content was determined following acid hydrolysis of the samples to convert polymeric carbohydrates into their corresponding monosaccharides. The resulting hydrolysates were subsequently analyzed using the phenol‑sulfuric acid colorimetric assay as previously described (Weinmann, 1947). Absorbance was measured at 490 nm using a UV–Vis spectrophotometer, and carbohydrate concentrations were quantified from a glucose calibration curve. The results were expressed as grams of glucose equivalents per 100 g fresh weight (g GE 100 g^−1^ FW). All analyses were performed in triplicate.

### 2.19 Total protein content

Total protein content was quantified using the Bradford colorimetric assay according to Bradford (1976). Briefly, an aliquot of the sample extract was mixed with Bradford reagent and incubated at room temperature to allow complete color development. The absorbance was subsequently measured at 595 nm using a UV–Vis spectrophotometer. A standard calibration curve was established using bovine serum albumin (BSA), and protein concentrations were calculated from the corresponding regression equation. The results were expressed as grams of BSA equivalents per 100 g fresh weight (g BSAE 100 g^−1^ FW) (Bradford, 1976).

### 2.20 Total phenolic content

Total phenolic content (TPC) was determined using the Folin-Ciocalteu colorimetric method with slight modifications. Briefly, 20 μL of appropriately diluted sample extract was mixed with 200 μL of tenfold diluted Folin-Ciocalteu reagent and 1.8 mL of distilled water. After 5–10 min of initial reaction at room temperature, 0.5 mL of sodium carbonate solution (20%, *w*/*v*) was added to neutralize the mixture and promote color development. The reaction mixture was vortexed thoroughly and incubated in the dark at room temperature for 30 min to allow complete chromophore formation. Absorbance was then measured at 765 nm using a UV–Vis spectrophotometer (Sánchez-Rangel et al., 2013). Gallic acid was used to construct a standard calibration curve, and total phenolic content was expressed as milligrams of gallic acid equivalents (GAE) per 100 g fresh weight (FW). All analyses were performed in triplicate to ensure analytical accuracy and reproducibility.

### 2.21 Antioxidant capacity

Antioxidant activity of the samples was evaluated using the 2,2-diphenyl-1-picrylhydrazyl (DPPH) free radical scavenging assay. Fresh tissue extracts were prepared by homogenizing the samples in 80% methanol, followed by centrifugation at 12,000 ×*g* for 15–20 min at 4 °C. The supernatant was collected and used for analysis (Marinova & Batchvarov, 2011). For the assay, 0.1 mL of sample extract was mixed with 3.9 mL of freshly prepared DPPH methanolic solution (0.025 g L^−1^). The reaction mixture was vortexed and incubated in the dark at room temperature for 10–30 min to allow the reaction between antioxidants and DPPH radicals. Absorbance was measured at 517 nm using a UV–Vis spectrophotometer. A control containing DPPH solution without sample extract was used as reference. All measurements were performed in triplicate.

Radical scavenging activity was calculated according to the following equation:

$$\text{DPPH scavenging activity}\;\left(\%\right)=\frac{A_{\mathrm{control}}-A_{\mathrm{sample}}}{A_{\mathrm{control}}}\times 100$$

where $A_{\mathrm{control}}$represents the absorbance of the DPPH solution without sample and $A_{\mathrm{sample}}$represents the absorbance in the presence of the extract.

### 2.22 Ascorbic acid content

Ascorbic acid content was determined using a commercial enzymatic assay kit according to the manufacturer's instructions. Briefly, approximately 300 mg of fresh tissue was homogenized in 500 μL of cold extraction (lysis) buffer to prevent oxidation during sample preparation. The homogenate was centrifuged at 15,000 ×*g* for 10 min at 4 °C to obtain a clear supernatant.

An appropriate aliquot of the supernatant was mixed with the kit's reaction reagents, including oxidizing and chromogenic solutions, to initiate color development. The reaction mixture was incubated at room temperature for the specified time to ensure complete conversion of ascorbic acid to the detectable product. Absorbance was measured at 500–540 nm using a UV–Vis spectrophotometer (Lee et al., 1997). Ascorbic acid concentration was calculated based on a standard calibration curve prepared using known concentrations of l-ascorbic acid and expressed as mg per 100 g fresh weight (FW). All analyses were performed in triplicate to ensure accuracy and reproducibility.

### 2.23 Electrolyte leakage

Electrolyte leakage (EL) was determined according to the method of Whitlow et al. (1992) with minor modifications. Briefly, approximately 0.2 g of fresh aril tissue was immersed in 10 mL of deionized water and incubated at room temperature for 24–26 h to allow diffusion of electrolytes from damaged cells. The initial electrical conductivity (EC₁) of the bathing solution was measured using a conductivity meter (Whitlow et al., 1992). The samples were subsequently boiled for 15 min to release the total cellular electrolytes, cooled to room temperature, and the final electrical conductivity (EC₂) was recorded. Electrolyte leakage was calculated using the following equation:

Electrolyte leakage was calculated as:

$$\mathrm{EL}\;\left(\%\right)=\frac{EC_{1}}{EC_{2}}\times 100$$

where *EC₁* represents the conductivity of electrolytes leaked from partially damaged cells and *EC₂* corresponds to the total electrolyte content released after boiling. All measurements were performed in triplicate and expressed as percentage electrolyte leakage.

### 2.24 Enzymatic activity

#### 2.24.1 Enzyme extraction

Frozen pomegranate aril samples were immediately pulverized to a fine powder in liquid nitrogen using a pre-chilled mortar and pestle to minimize enzymatic degradation. Approximately 2 g of powdered tissue was homogenized in ice-cold extraction buffer consisting of 50 mM potassium phosphate buffer (pH 7.0–7.5), 1 mM ethylenediaminetetraacetic acid (EDTA), and 1–3 mM MgCl₂ under chilled conditions. The homogenate was centrifuged at 20,000–25,000 ×g for 20 min at 4 °C, and the resulting supernatant was carefully collected and used as the crude enzyme extract for subsequent enzymatic analyses. All extraction procedures were performed at 4 °C to maintain enzyme stability and preserve catalytic activity.

#### 2.24.2 Catalase (CAT)

Catalase (EC 1.11.1.6) activity was determined according to the method of Aebi (1984) by monitoring the decomposition of hydrogen peroxide (H₂O₂). The reaction mixture consisted of 50 mM potassium phosphate buffer (pH 7.4), 10 mM H₂O₂, and an aliquot of crude enzyme extract. The decrease in absorbance at 240 nm resulting from H₂O₂ consumption was continuously recorded for 1–2 min using a UV–Vis spectrophotometer. One unit (U) of CAT activity was defined as the amount of enzyme catalyzing the decomposition of 1 μmol of H₂O₂ min^−1^ under the assay conditions (Aebi, 1984). Enzyme activity was expressed as U g^−1^ fresh weight (FW), and all measurements were performed in triplicate.

#### 2.24.3 Superoxide dismutase (SOD)

Superoxide dismutase (EC 1.15.1.1) activity was determined according to the method of Beauchamp and Fridovich (1971) based on the enzyme-mediated inhibition of nitro blue tetrazolium (NBT) photoreduction. The reaction mixture contained 50 mM potassium phosphate buffer (pH 7.8), methionine, NBT, riboflavin, EDTA, and an aliquot of crude enzyme extract. The reaction mixtures were exposed to fluorescent light for 10 min to initiate NBT photoreduction, after which absorbance was measured at 560 nm using a UV–Vis spectrophotometer (Beauchamp & Fridovich, 1971). One unit (U) of SOD activity was defined as the amount of enzyme required to cause 50% inhibition of NBT photoreduction under the assay conditions. Enzyme activity was expressed as U g^−1^ fresh weight (FW), and all measurements were conducted in triplicate.

#### 2.24.4 Peroxidase (POD)

Peroxidase (EC 1.11.1.7) activity was determined according to the method of Chance and Maehly (1955) using *o*-dianisidine as the hydrogen donor substrate in the presence of hydrogen peroxide (H₂O₂). The reaction mixture consisted of phosphate buffer (pH 6.5), *o*-dianisidine, H₂O₂, and an aliquot of crude enzyme extract. The enzymatic oxidation of *o*-dianisidine was monitored spectrophotometrically by recording the increase in absorbance at 485 nm using a UV–Vis spectrophotometer (Chance & Maehly, 1955). One unit (U) of POD activity was defined as the amount of enzyme causing an increase of one absorbance unit min^−1^ under the assay conditions. Enzyme activity was expressed as U g^−1^ fresh weight (FW), and all measurements were performed in triplicate.

#### 2.24.5 Polyphenol oxidase (PPO)

Polyphenol oxidase (EC 1.14.18.1) activity was determined according to the method of Mayer and Harel (1979) using catechol as the substrate. The reaction mixture consisted of sodium phosphate buffer (pH 6.5), catechol solution, and an aliquot of crude enzyme extract. The enzymatic oxidation of catechol to quinone products was monitored spectrophotometrically by measuring the increase in absorbance at 420 nm using a UV–Vis spectrophotometer (Mayer & Harel, 1979). One unit (U) of PPO activity was defined as the amount of enzyme causing an increase of 1.0 absorbance unit min^−1^ under the assay conditions. Enzyme activity was expressed as U g^−1^ fresh weight (FW), and all analyses were performed in triplicate.

#### 2.24.6 Phenylalanine ammonia-lyase (PAL)

Phenylalanine ammonia-lyase (EC 4.3.1.24) activity was determined according to the method of Friend et al. (1973) by quantifying the formation of trans-cinnamic acid from *L*-phenylalanine. The reaction mixture consisted of *L*-phenylalanine solution, Tris-HCl buffer (pH 8.8), and an aliquot of crude enzyme extract and was incubated at 37 °C for 60 min. The reaction was terminated by the addition of hydrochloric acid, and the formation of trans-cinnamic acid was monitored spectrophotometrically by measuring the absorbance at 290 nm (Friend et al., 1973). One unit (U) of PAL activity was defined as the amount of enzyme producing 1 μmol of trans-cinnamic acid min^−1^ under the assay conditions. Enzyme activity was expressed as U g^−1^ fresh weight (FW), and all measurements were performed in triplicate.

### 2.25 Microbiological analysis

Microbiological analyses were conducted aseptically throughout refrigerated storage. For each of three independent biological replicates, 10 g of pomegranate arils was transferred to a sterile stomacher bag containing 90 mL of sterile 0.1% (*w*/*v*) peptone water and homogenized for 2–3 min. Serial tenfold dilutions were prepared in the same diluent. For each dilution, 0.1 mL was surface-plated in duplicate. Total mesophilic aerobic bacteria were enumerated on Plate Count Agar after incubation at 37 ± 1 °C for 48 h, whereas yeasts and molds were enumerated on Potato Dextrose Agar supplemented with chloramphenicol after incubation at 28 ± 1 °C for 3–5 days. Plates containing 30–300 colonies were used for quantitative enumeration, and results were expressed as log CFU g^−1^. Based on the initial 10^−1^ homogenate and a combined duplicate-plating volume of 0.2 mL, the method detection limit was 50 CFU g^−1^ (1.70 log CFU g^−1^). Samples yielding no colonies were therefore reported as <1.70 log CFU g^−1^, while plates containing fewer than 30 colonies were considered estimated counts below the reliable quantification range (Lampel et al., 2001).

### 2.26 GC–MS analysis of Persian hogweed essential oil

The volatile composition of Persian hogweed EO was characterized by gas chromatography–mass spectrometry (GC–MS). Analyses were performed using a gas chromatograph coupled to a mass selective detector equipped with a fused-silica capillary column (30 m × 0.25 mm i.d., 0.25 μm film thickness). Helium was employed as the carrier gas at a constant flow rate of 1.0 mL min^−1^. The injector temperature was maintained at 250 °C, and samples were introduced in split mode. The oven temperature program was initiated at 50 °C and held for 3 min, followed by heating at a rate of 5 °C min^−1^ to 250 °C, where it was maintained for 5 min to ensure complete elution of volatile constituents. The mass spectrometer was operated in electron impact (EI) ionization mode at 70 eV, and mass spectra were acquired over the range of *m*/*z* 40–500.

Identification of EO constituents was achieved by comparison of the acquired mass spectra with those contained in the NIST mass spectral database and by calculation of retention indices (RI) relative to a homologous series of *n*-alkanes analyzed under identical chromatographic conditions. Relative abundances of individual compounds were estimated by peak area normalization without correction factors and expressed as percentages of the total ion chromatogram (TIC). Only compounds exhibiting spectral similarity indices ≥90% and consistent retention indices were considered positively identified.

### 2.27 In vitro release of EO from electrospun microfibers

The release of Persian hogweed EO from EO-loaded and nanoencapsulated-EO-loaded PVA/CH microfibers was evaluated under simulated refrigerated-package conditions. Microfiber specimens containing equivalent initial amounts of EO were placed in sealed 100-mL amber glass containers above 10 mL of distilled water without direct contact with the liquid. The water maintained a high relative humidity similar to that surrounding packaged fresh-cut arils. Containers were stored at 4 ± 1 °C, and samples were collected after 0, 1, 3, 6, 12, 24, 48, 72, 96, and 120 h. Three independent containers were prepared for each formulation and sampling time.

At each interval, the microfiber specimen was removed and the residual EO was extracted using 10 mL of n-hexane under sonication for 30 min. After centrifugation at 10,000 ×*g* for 10 min, the EO concentration in the supernatant was determined by gas chromatography-flame ionization detection. The principal compound identified during the GC–MS spectrometry analysis of Persian hogweed EO was used as the quantitative marker. Quantification was performed using an external calibration curve prepared from known concentrations of the EO. Blank PVA/CH microfibers were analyzed under identical conditions to correct for matrix interference. The initial EO loading was expressed as milligrams of EO per gram of dry microfiber.

Cumulative EO release was calculated from the reduction in the residual oil content of the microfibers:

$$\text{Cumulative release}\;\left(\%\right)=\frac{M_{0}-M_{t}}{M_{0}}\times 100$$

where is the initial amount of EO incorporated into the microfiber and is the amount remaining at time. Release measurements were expressed as mean ± standard deviation of three independent replicates. The release data were fitted to zero-order, first-order, Higuchi, and Korsmeyer-Peppas models. Model suitability was evaluated using the coefficient of determination and root-mean-square error.

### 2.28 Statistical analysis

All data were analyzed according to a two-factor factorial design using two-way analysis of variance to determine the main effects of treatment and storage period and their interaction. Statistical analyses were performed using SAS software (version 9.1; SAS Institute Inc., Cary, NC, USA). Before analysis, normality of residuals and homogeneity of variances were evaluated using the Shapiro-Wilk and Levene's tests, respectively. Data were transformed when necessary to satisfy the assumptions of analysis of variance. When a significant treatment × storage-period interaction was detected, simple-effect comparisons were performed using Duncan's multiple range test at *p* < 0.05. Treatments were compared separately within each storage period, and storage periods were compared separately within each treatment. In the tables, different lowercase letters indicate significant differences among treatments within the same storage period, whereas different uppercase letters indicate significant differences among storage periods within the same treatment. Means sharing at least one letter are not significantly different. Results are expressed as mean ± standard error of three independent replicates (*n* = 3). Figures were prepared using Microsoft Excel.

## 3 Result

### 3.1 FTIR analysis of carbon dots

The surface chemistry of the CDs was investigated by FTIR spectroscopy over the spectral range of 4000–400 cm^−1^ (Fig. 1A). The FTIR spectrum exhibited several characteristic absorption bands, confirming the enrichment of the CD surface with oxygen- and nitrogen-containing functionalities. A broad and intense band centered at approximately 3440 cm^−1^ (3600–3200 cm^−1^) was assigned to O—H stretching vibrations associated with hydroxyl groups and adsorbed water molecules. The considerable broadening of this band suggests extensive intermolecular hydrogen-bonding interactions, reflecting the hydrophilic nature of the CDs and their favorable dispersibility in aqueous media.

**Fig. 1:**
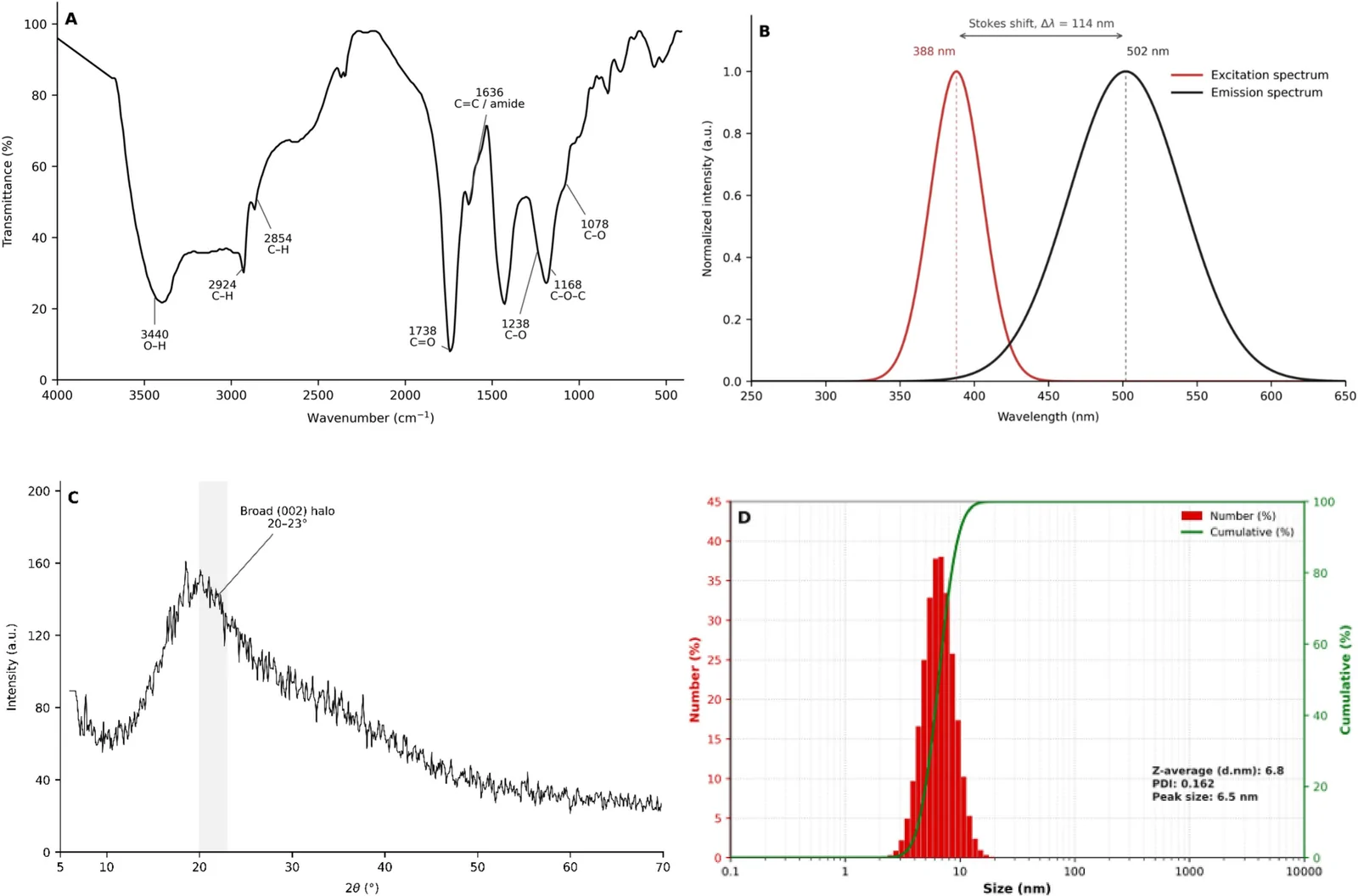
Structural, optical, and particle-size characterization of the synthesized carbon dots. (A) Fourier-transform infrared spectrum showing the characteristic surface functional groups and chemical bonds. (B) Photoluminescence emission spectrum demonstrating their optical behavior. (C) X-ray diffraction pattern showing the broad diffraction halo characteristic of predominantly amorphous carbon. (D) Dynamic light-scattering profile showing the number-weighted hydrodynamic particle-size distribution and cumulative size distribution of the carbon dots.

Weak absorption bands at 2924 and 2854 cm^−1^ were attributed to asymmetric and symmetric stretching vibrations of aliphatic C—H bonds originating from residual -CH₂ and -CH₃ moieties. A pronounced absorption peak located at 1738 cm^−1^ was assigned to the stretching vibration of carbonyl (C=O) groups, likely associated with carboxylic acid, ester, or lactone functionalities generated during the carbonization and surface oxidation processes. In addition, the band detected at approximately 1636 cm^−1^ may be attributed to C=C stretching vibrations of sp^2^-hybridized aromatic domains and/or amide-related groups, indicating the coexistence of conjugated graphitic structures and surface heteroatom functionalities within the carbonaceous framework.

Strong absorption bands appearing within the 1200–1000 cm^−1^ region, particularly at 1238, 1168, and 1078 cm^−1^, were assigned to C—O and C-O-C stretching vibrations, confirming the abundance of hydroxyl, ether, and carboxyl-derived surface groups. These oxygenated functionalities are expected to enhance the colloidal stability, redox activity, and interaction potential of CDs with polymeric matrices. Several low-intensity bands detected in the 900–500 cm^−1^ region were tentatively assigned to out-of-plane aromatic C—H bending modes and skeletal vibrations of disordered carbon domains, further supporting the formation of a partially graphitized yet predominantly functionalized carbon nanostructure.

### 3.2 Photoluminescence properties of carbon dots

The optical properties of the CDs were evaluated by UV–Vis absorption and PL spectroscopy (Fig. 1B). The CDs exhibited a pronounced absorption maximum at 388 nm, which is commonly attributed to n-π* transitions associated with surface carbonyl functionalities and conjugated domains. Upon excitation at 388 nm, the nanoparticles generated a strong emission band centered at 502 nm within the green region of the visible spectrum. The emission peak was relatively narrow and symmetric, suggesting a reasonably uniform distribution of emissive surface states and good optical homogeneity of the CDs.

The distinct separation between the excitation and emission maxima resulted in a Stokes shift of approximately 114 nm, indicative of reduced spectral overlap and diminished self-absorption effects, thereby favoring efficient fluorescence emission. The intense green fluorescence observed at 502 nm is likely associated with radiative recombination processes occurring through surface defect sites and oxygen-containing functional groups, which act as emissive centers. This interpretation is supported by FTIR analysis, which revealed abundant hydroxyl, carbonyl, and ether functionalities decorating the CD surface. The well-resolved excitation and emission features demonstrate that the CDs possess stable photoluminescent characteristics and favorable optical properties. Moreover, the relatively large Stokes shift may be advantageous for applications requiring high fluorescence contrast, including bioimaging, chemical sensing, and photoactivated antimicrobial systems.

### 3.3 X-ray diffraction (XRD)

The crystalline structure of the CDs was examined by XRD analysis (Fig. 1C). The diffraction pattern exhibited a broad peak centered at approximately 2θ = 20–23°, indicating the predominantly amorphous character of the carbon nanomaterial. The absence of sharp and well-resolved diffraction peaks suggests a lack of extensive long-range crystallographic ordering within the carbon framework. This broad diffraction halo is commonly assigned to the (002) plane of disordered graphitic carbon and is indicative of turbostratic carbon domains possessing only short-range structural organization. Such diffraction behavior is characteristic of CDs and other partially graphitized carbonaceous nanostructures. No additional diffraction peaks attributable to crystalline impurities or secondary phases were detected within the scanned 2θ range of 5–70°, implying that the synthesized material was largely composed of amorphous carbon species. Furthermore, the progressive attenuation of diffraction intensity at higher angles is consistent with a highly disordered carbon matrix.

### 3.4 Dynamic light-scattering analysis

Dynamic light-scattering analysis showed a narrow, unimodal size distribution for the carbon dots, with a peak hydrodynamic diameter of approximately 6.5 nm and a *Z*-average diameter of 6.8 nm (Fig. 1D). The polydispersity index was 0.162, indicating a relatively uniform particle population and good dispersion stability with limited aggregation. These results confirm the nanoscale dimensions and homogeneous hydrodynamic behavior of the synthesized carbon dots.

### 3.5 Morphological evaluation of the electrospun microfiber

The surface morphology of the electrospun microfibrous mats was examined by SEM (Fig. 2A-D). The control microfiber mat, fabricated from a 10% PVA/5% CH blend, exhibited a continuous and randomly oriented fibrous network composed of smooth, bead-free fibers, indicating adequate polymer chain entanglement, favorable solution properties, and stable electrospinning conditions (Fig. 2A).

**Fig. 2:**
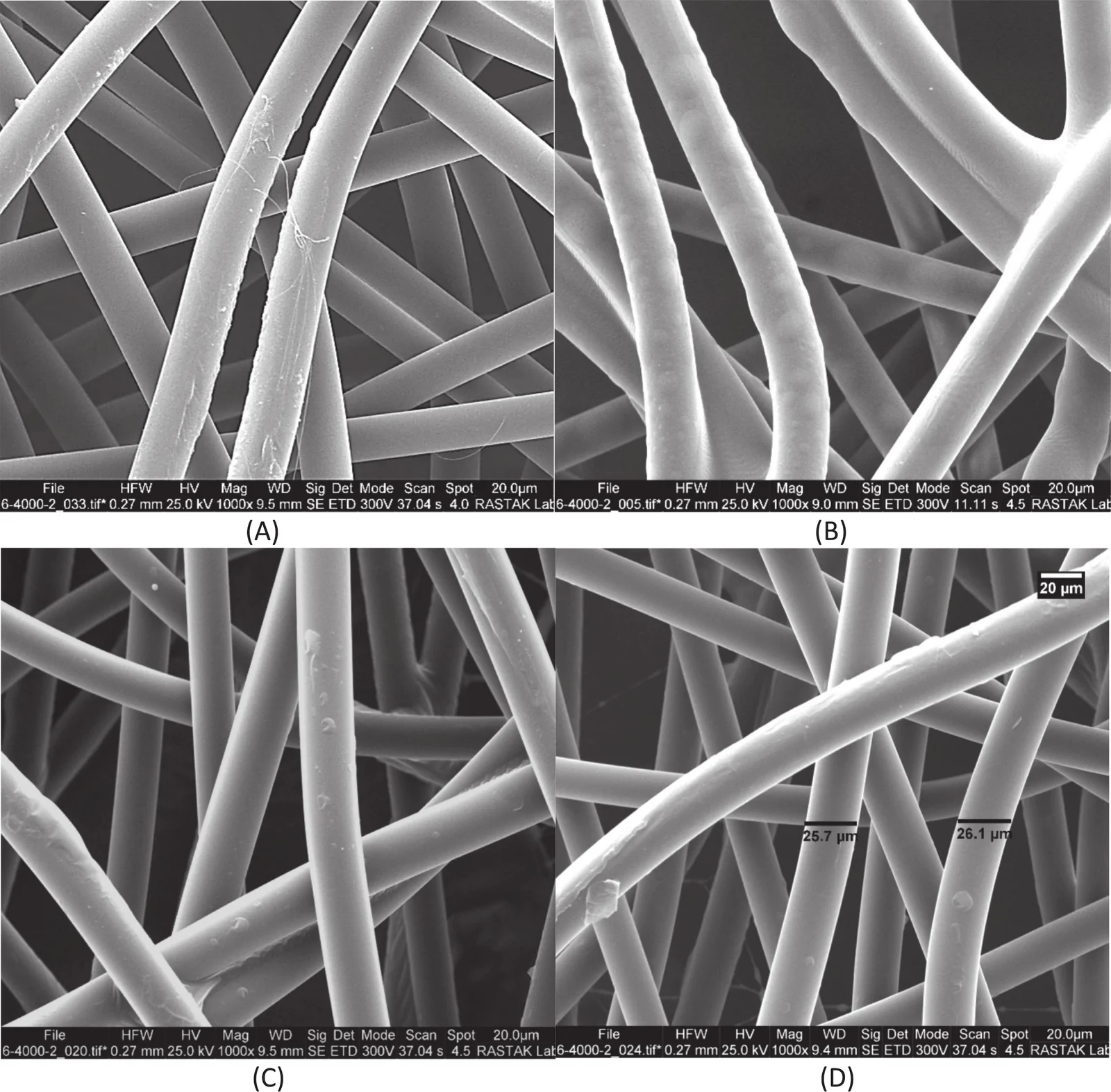
Morphological characterization of the electrospun microfiber pads: (A) control microfiber mat composed of 10% polyvinyl alcohol and 5% chitosan; (B) polyvinyl alcohol/chitosan microfiber pad containing carbon dots; (C) polyvinyl alcohol/chitosan microfiber pad containing free Persian hogweed (*Heracleum persicum*) essential oil; and (D) polyvinyl alcohol/chitosan microfiber pad containing nanoencapsulated Persian hogweed essential oil.

Incorporation of CD-loaded chitosan nanocarriers did not substantially alter the overall fibrous architecture, although a slightly roughened fiber surface was observed (Fig. 2B). This morphological feature may be attributed to the presence of embedded nanocarriers and suggests effective distribution of CDs throughout the polymeric matrix. Importantly, the absence of significant fiber defects or bead formation indicates that the addition of CDs had a negligible impact on jet stability and fiber formation during electrospinning, reflecting favorable interactions between the nanocarriers and the PVA/CH matrix.

The microfibrous mat containing free Persian hogweed EO exhibited noticeable morphological alterations compared with the control, including localized bead formation and increased heterogeneity in fiber appearance (Fig. 2C). These structural irregularities are likely associated with modifications in solution viscosity, conductivity, and surface tension induced by the direct incorporation of the hydrophobic EO, which may adversely affect jet stretching and solvent evaporation dynamics during electrospinning. In contrast, microfibers incorporating EONCs displayed a more uniform, continuous, and bead-free morphology than those containing free EO (Fig. 2D).

### 3.6 Weight loss

Weight loss increased progressively during refrigerated storage in all treatments; however, its magnitude differed significantly among the packaging systems. After 20 days, control arils exhibited the highest weight loss (0.788%), followed by those treated with free EO (0.363%). Carbon-dot and nanoencapsulated-EO treatments significantly limited weight loss to 0.318% and 0.303%, respectively, with the nanoencapsulated formulation providing the greatest protection against moisture loss (Table 1).

**Table 1:** Interaction effect of treatment and storage time on physicochemical and color parameters.

Treatment	Day	Weight loss (%)	a*	b*	L*	C*	h°	pH	Acidity
Control	1	0.000 ± 0.000 o	8.55 ± 0.028 de	3.85 ± 0.031 f	10.13 ± 0.014 bc	9.34 ± 0.12 g	26.45 ± 0.026 ab	3.54 ± 0.011 i	1.19 ± 0.052 a
	5	0.145 ± 0.003 l	8.42 ± 0.013 h	4.01 ± 0.008 cd	10.32 ± 0.010 ab	9.31 ± 0.12 g	25.63 ± 1.22 d	3.89 ± 0.007 f	0.83 ± 0.066 d
	10	0.383 ± 0.001 c	8.32 ± 0.009 j	3.81 ± 0.017 g	10.22 ± 0.012 bc	9.14 ± 0.45 i	24.71 ± 2.008 f	3.96 ± 0.028 e	0.78 ± 0.047 e
	15	0.668 ± 0.001 b	8.66 ± 0.010 c	4.16 ± 0.019 b	10.13 ± 0.124 bc	9.80 ± 0.02 b	25.66 ± 0.58 cd	4.12 ± 0.009 c	0.43 ± 0.028 h
	20	0.788 ± 0.001 a	8.86 ± 0.011 b	4.26 ± 0.010 a	9.03 ± 0.45 h	9.83 ± 0.102 b	25.68 ± 0.987 cd	4.27 ± 0.010 a	0.35 ± 0.089 i
Carbon dots (CDs)	1	0.000 ± 0.000 o	8.55 ± 0.027 de	3.85 ± 0.029 f	10.13 ± 0.013 bc	9.34 ± 0.17 g	26.45 ± 0.025 ab	3.54 ± 0.012 i	1.19 ± 0.076 a
	5	0.018 ± 0.001 n	8.46 ± 0.009 g	3.98 ± 0.068 d	10.47 ± 0.011 a	9.33 ± 0.97 g	25.19 ± 0.125 e	3.76 ± 0.008 h	0.96 ± 0.039 c
	10	0.158 ± 0.001 k	8.36 ± 0.128 i	3.87 ± 0.057 f	10.31 ± 0.78 ab	9.22 ± 0.007 h	24.71 ± 0.365 f	3.84 ± 0.107 g	0.86 ± 0.018 d
	15	0.290 ± 0.003 g	8.57 ± 0.109 d	4.05 ± 0.012 c	10.16 ± 0.36 bc	9.51 ± 0.02 e	25.65 ± 0.630 cd	3.93 ± 0.008 f	0.72 ± 0.121 f
	20	0.318 ± 0.001 e	8.78 ± 0.010 b	4.19 ± 0.02 b	9.56 ± 0.74 g	9.73 ± 0.10 c	25.62 ± 0.12 cd	3.97 ± 0.014 e	0.54 ± 0.010 g
Essential oil (EO)	1	0.000 ± 0.000 o	8.55 ± 0.026 de	3.85 ± 0.030 f	10.13 ± 0.112 bc	9.34 ± 0.023 g	26.45 ± 2.026 ab	3.54 ± 0.014 i	1.19 ± 0.037 a
	5	0.048 ± 0.001 m	8.38 ± 0.010 h	3.93 ± 0.089 e	10.34 ± 0.74 ab	9.25 ± 0.109 h	25.18 ± 0.008 e	3.73 ± 0.007 h	0.95 ± 0.023 c
	10	0.185 ± 0.003 i	8.40 ± 0.209 h	3.83 ± 0.018 g	10.12 ± 0.01 c	9.23 ± 0.895 h	25.71 ± 2.05 c	3.94 ± 0.028 f	0.69 ± 0.009 f
	15	0.308 ± 0.001 f	8.56 ± 0.810 de	4.13 ± 0.089 b	9.81 ± 0.125 e	9.50 ± 0.781 e	25.66 ± 2.009 cd	4.04 ± 0.010 d	0.54 ± 0.022 g
	20	0.363 ± 0.001 d	8.77 ± 0.009 b	3.89 ± 0.058 f	9.04 ± 0.78 h	9.59 ± 0.326 d	23.75 ± 0.010 g	4.17 ± 0.011 b	0.42 ± 0.000 h
Nanoencapsulated EO (EONCs)	1	0.000 ± 0.000 o	8.55 ± 0.127 de	3.85 ± 0.028 f	10.13 ± 0.456 bc	9.34 ± 0.854 g	26.45 ± 0.024 ab	3.54 ± 0.010 i	1.19 ± 0.006 a
	5	0.023 ± 0.001 n	8.38 ± 0.809 h	3.97 ± 0.002 d	10.33 ± 0.149 ab	9.27 ± 0.120 h	27.19 ± 0.095 a	3.73 ± 0.018 h	0.94 ± 0.008 c
	10	0.170 ± 0.000 j	8.45 ± 0.008 g	3.70 ± 0.04 h	10.18 ± 0.256 bc	9.22 ± 0.021 h	23.75 ± 0.009 g	3.84 ± 0.36 g	0.76 ± 0.008 e
	15	0.278 ± 0.001 h	8.48 ± 0.229 f	4.21 ± 0.085 b	10.03 ± 0.741 cd	9.46 ± 0.007 f	26.57 ± 0.98 ab	3.96 ± 0.010 e	0.65 ± 0.009 f
	20	0.303 ± 0.001 f	8.90 ± 0.011 a	4.32 ± 0.010 a	9.22 ± 0.008 f	9.89 ± 0.009 a	25.66 ± 0.458 cd	4.07 ± 0.011 d	0.57 ± 0.010 g

Values are presented as mean ± SE (*n* = 3). Within each column, means followed by different lowercase letters are significantly different according to Duncan's multiple range test (*p* < 0.05).

### 3.7 pH and acidity

The pH values increased progressively during refrigerated storage in all treatments, concomitant with a decline in TA, although the magnitude of these changes varied significantly among packaging systems (Table 1). Control samples exhibited the most pronounced increase in pH, reaching 4.27 after 20 days of storage, whereas CDs-treated arils maintained significantly lower pH values (3.97) throughout the storage period. Similarly, TA decreased markedly in control samples, declining to 0.35%, while arils treated with CDs and EONCs retained significantly higher acidity levels.

### 3.8 Color parameters *(L, a*, b*, C*, h°)

Color parameters were significantly influenced by both packaging treatment and storage duration, as evidenced by a significant treatment × storage interaction (Table 1). The lightness parameter (L*) progressively declined during storage in all treatments, reflecting the gradual loss of visual freshness and pigment stability. However, the reduction was more pronounced in Control and free EO-treated samples, which exhibited the lowest L* values at day 20 (9.03 and 9.04, respectively). In contrast, CDs-treated arils maintained significantly higher L* values (9.56), suggesting improved preservation of tissue brightness and reduced browning-related discoloration.

The redness parameter (a*) increased throughout storage, with EONCs-treated samples exhibiting the highest value (8.90) at day 20. Likewise, yellowness (b*) and chroma (C*) increased significantly during storage, reaching maximum values of 4.32 and 9.89, respectively, in EONCs-treated arils. The elevated chroma values indicate enhanced color saturation and retention of visual intensity. Nevertheless, the concurrent increase in b* values may reflect complex pigment transformations occurring during storage, including anthocyanin degradation, copigmentation phenomena, or the formation of non-enzymatic browning products. The hue angle (h°) exhibited relatively minor fluctuations among treatments, although control samples showed a more pronounced decline over time, indicating greater alterations in overall color tone.

### 3.9 Total soluble solids

Total soluble solids declined progressively during refrigerated storage in all treatments, reflecting the utilization of soluble carbohydrates and other low-molecular-weight metabolites as respiratory substrates during senescence (Table 2). However, the magnitude of TSS depletion varied significantly among packaging treatments. Control arils exhibited the greatest reduction in TSS, decreasing from 16.65°Brix on day 1 to 15.46°Brix on day 20, corresponding to a loss of approximately 7.1%. In contrast, CDs-treated samples retained significantly higher TSS values throughout storage, reaching 16.04°Brix at day 20, followed by EONCs (15.84°Brix), whereas free EO-treated arils showed intermediate retention (15.63°Brix). The significant treatment × storage interaction indicates that nano-enabled packaging systems were particularly effective in limiting soluble solid depletion during the later stages of storage (days 15–20), when senescence-associated metabolic activity became more pronounced. The superior preservation of TSS in CDs- and EONCs-treated arils may be attributed to the attenuation of respiratory metabolism, maintenance of cellular compartmentalization, and suppression of oxidative and microbial deterioration.

**Table 2:** Interaction effect of treatment and storage time on biochemical attributes.

Treatment	Day	TSS (°Brix)	Firmness (N)	Total sugar (g 100 g^−1^ FW)	Total phenol (mg GAE 100 g^−1^ FW)	Antioxidant capacity (%)	Total carbohydrates (g 100 g^−1^ FW)	Total protein (g 100 g^−1^ FW)
Control	1	16.645 ± 0.258 a	8.250 ± 0.029 a	13.165 ± 0.010 a	297.985 ± 1.10 a	50.410 ± 0.012 a	9.500 ± 0.145 g	13.775 ± 0.15 g
	5	16.270 ± 0.456 d	7.850 ± 0.015 c	12.440 ± 0.006 c	271.570 ± 2.30 d	47.305 ± 0.985 d	9.875 ± 0.265 f	13.715 ± 0.054 h
	10	16.150 ± 0.784 f	6.250 ± 0.015 f	10.485 ± 0.013 f	233.205 ± 8.60 g	44.760 ± 1.015 f	10.130 ± 0.012 e	13.685 ± 0.110 i
	15	15.890 ± 0.895 h	5.550 ± 0.015 h	8.210 ± 0.012 i	212.895 ± 0.60 i	37.340 ± 0.012 i	10.315 ± 0.74 d	13.675 ± 0.124 i
	20	15.460 ± 0.470 k	4.500 ± 0.058 j	5.770 ± 0.082 l	188.355 ± 8.00 l	25.205 ± 0.015 l	10.880 ± 0.126 a	13.955 ± 0.015 d
Carbon dots (CDs)	1	16.645 ± 0.014 a	8.250 ± 0.029 a	13.165 ± 0.010 a	297.985 ± 1.10 a	50.410 ± 0.012 a	9.500 ± 0.987 g	13.775 ± 0.114 g
	5	16.615 ± 0.145 b	8.150 ± 0.015 b	12.545 ± 0.023 b	284.575 ± 6.60 b	48.845 ± 0.236 b	9.690 ± 0.103 f	13.780 ± 0.223 g
	10	16.610 ± 0.789 b	7.600 ± 0.020 d	11.345 ± 0.045 d	257.575 ± 0.70 e	46.790 ± 0.010 e	9.915 ± 0.004 e	13.710 ± 0.012 h
	15	16.305 ± 0.032 c	6.900 ± 0.079 e	10.230 ± 0.112 g	235.420 ± 2.80 g	42.965 ± 0.012 g	10.065 ± 0.184 e	13.655 ± 0.784 i
	20	16.035 ± 0.143 e	5.300 ± 0.019 i	8.580 ± 0.109 j	212.445 ± 2.80 i	39.580 ± 0.741 h	10.565 ± 1.009 c	13.995 ± 0.012 c
Essential oil (EO)	1	16.645 ± 0.120 a	8.250 ± 0.029 a	13.165 ± 0.120 a	297.985 ± 1.10 a	50.410 ± 0.012 a	9.500 ± 0.006 g	13.775 ± 0.324 g
	5	16.635 ± 0.178 b	7.300 ± 0.058 g	12.460 ± 0.206 c	290.160 ± 5.50 b	49.225 ± 0.985 b	9.580 ± 0.032 g	13.950 ± 0.114 d
	10	16.575 ± 0.236 c	6.850 ± 0.015 e	10.810 ± 0.62 e	265.450 ± 0.70 d	48.700 ± 1.012 c	9.760 ± 0.874 f	13.895 ± 0.021 e
	15	16.080 ± 0.784 e	5.800 ± 0.036 g	9.415 ± 0.450 h	242.690 ± 0.60 f	43.895 ± 0.012 f	9.935 ± 0.009 e	13.830 ± 0.014 f
	20	15.630 ± 0.136 j	5.000 ± 0.154 i	5.395 ± 0.020 m	222.265 ± 5.80 h	38.065 ± 0.015 i	10.445 ± 0.112 c	14.135 ± 0.110 a
Nanoencapsulated EO (EONCs)	1	16.645 ± 0.032 a	8.250 ± 0.129 a	13.165 ± 0.010 a	297.985 ± 1.10 a	50.410 ± 0.012 a	9.500 ± 0.006 g	13.775 ± 0.951 g
	5	16.615 ± 0.730 b	7.350 ± 0.15 g	12.505 ± 0.76 b	281.830 ± 0.50 c	48.210 ± 0.732 c	9.610 ± 0.225 g	13.785 ± 0.143 g
	10	16.475 ± 0.125 d	7.050 ± 0.015 d	11.040 ± 0.032 d	257.210 ± 2.60 e	47.955 ± 0.010 d	9.710 ± 0.111 f	13.720 ± 0.086 h
	15	16.255 ± 0.146 f	6.450 ± 0.078 f	10.195 ± 0.485 g	241.995 ± 6.40 f	44.475 ± 0.032 e	9.865 ± 0.147 e	13.675 ± 0.026 i
	20	15.835 ± 0.010 i	4.950 ± 0.025 j	8.975 ± 0.703 k	231.340 ± 1.80 g	41.610 ± 0.145 g	10.120 ± 0.326 e	13.975 ± 0.010 c

Values are presented as mean ± SE (*n* = 3). Within each column, means followed by different lowercase letters are significantly different according to Duncan's multiple range test (*p* < 0.05).

### 3.10 Fruit firmness

Fruit firmness decreased progressively during refrigerated storage in all treatments, although the extent of tissue softening differed significantly among packaging systems (Table 2). Control arils exhibited the most pronounced loss of firmness, declining from 8.25 N at day 1 to 4.50 N at day 20, corresponding to an overall reduction of approximately 45%. In contrast, CDs-treated samples maintained significantly higher firmness values throughout storage, reaching 5.30 N at day 20, followed by EO (5.00 N) and EONCs (4.95 N). Statistically significant differences among treatments became evident from day 10 onward, indicating that nano-enabled packaging effectively delayed texture deterioration during extended refrigerated storage.

### 3.11 Total sugar

Total sugar content decreased significantly during refrigerated storage in all treatments, with a pronounced treatment × storage interaction effect (Table 2). Control arils exhibited the most substantial decline, reaching 5.77 g 100 g^−1^ FW after 20 days of storage, corresponding to an overall reduction of approximately 56% relative to the initial value. Similarly, free EO-treated samples experienced considerable sugar depletion, declining to 5.40 g 100 g^−1^ FW at day 20. In contrast, CDs- and EONCs-treated arils retained significantly higher sugar contents throughout storage, reaching 8.58 and 8.98 g 100 g^−1^ FW, respectively, at the end of the storage period.

### 3.12 Total phenolic content

Total phenolic content declined progressively throughout refrigerated storage in all treatments, although the extent of degradation varied significantly among packaging systems (Table 2). Control arils exhibited the most pronounced reduction, decreasing from 297.99 to 188.36 mg GAE 100 g^−1^ FW after 20 days of storage, corresponding to an overall loss of approximately 37%. In contrast, EONCs-treated samples retained significantly higher phenolic concentrations at the end of storage (231.34 mg GAE 100 g^−1^ FW), followed by EO- (222.27 mg GAE 100 g^−1^ FW) and CDs-treated arils (212.45 mg GAE 100 g^−1^ FW). Significant divergence between treated and control samples became evident from day 10 onward, demonstrating the effectiveness of nano-enabled packaging in mitigating phenolic degradation during extended refrigerated storage.

### 3.13 Antioxidant capacity

Antioxidant capacity declined progressively during refrigerated storage in all treatments, closely paralleling the trend observed for total phenolic content, thereby highlighting the major contribution of phenolic compounds to the overall antioxidant potential of pomegranate arils (Table 2). Control samples exhibited the most pronounced decrease, declining from 50.41% to 25.21% after 20 days of storage, corresponding to an overall reduction of approximately 50%. In contrast, EONCs-treated arils retained significantly higher antioxidant activity (41.61%) at the end of storage, followed by CDs (39.58%) and free EO-treated samples (38.07%). Differences among treatments became particularly pronounced after day 15, indicating that nano-enabled packaging systems were more effective in preserving antioxidant functionality during the later stages of storage.

### 3.14 Total carbohydrates

Total carbohydrate content exhibited a slight but significant increase during refrigerated storage, with the most pronounced rise observed in Control samples, increasing from 9.50 to 10.88 g 100 g^−1^ FW by day 20 (Table 2). In contrast, CDs- and EONCs-treated arils showed comparatively smaller fluctuations and maintained more stable carbohydrate concentrations throughout storage.

### 3.15 Total protein

Total protein content exhibited a slight but significant increase during refrigerated storage in all treatments, although the magnitude of change was less pronounced than that observed for sugars, phenolics, and antioxidant capacity (Table 2). Among the treatments, free EO-treated arils exhibited the highest protein concentration at day 20 (14.14 g 100 g^−1^ FW), followed closely by CDs (13.99 g 100 g^−1^ FW) and EONCs (13.98 g 100 g^−1^ FW). Control samples generally maintained lower protein levels, particularly during the early stages of storage.

### 3.16 Antioxidant enzymes

#### 3.16.1 Catalase

Catalase activity increased progressively during refrigerated storage in all treatments; however, the magnitude of induction differed significantly among packaging systems (Table 3). Control arils exhibited the most pronounced increase in CAT activity, rising from 8.47 U g^−1^ FW on day 1 to 11.10 U g^−1^ FW at day 20, thereby constituting the highest statistical group. In contrast, nano-treated samples maintained significantly lower CAT activities during the later stages of storage, with CDs, EO, and EONCs treatments reaching 9.09, 9.52, and 9.47 U g^−1^ FW, respectively, at day 20. Differences between control and treated samples became apparent from day 10 onward, coinciding with the onset of accelerated senescence and oxidative deterioration in untreated fruit.

**Table 3:** Interaction effect of treatment and storage time on enzymatic activity, microbial load and membrane stability.

Treatment	Day	Catalase activity (U g^−1^ FW)	Phenylalanine ammonia-lyase activity (U g^−1^ FW)	Peroxidase activity (U g^−1^ FW)	Polyphenol oxidase activity (U g^−1^ FW)	Superoxide dismutase activity (U g^−1^ FW)
Control	1	8.465 ± 0.009 j	43.185 ± 0.020 b	1.145 ± 0.003 i	25.285 ± 0.032 p	0.975 ± 0.003 k
	5	9.235 ± 0.009 f	43.135 ± 0.009 b	1.030 ± 0.012 l	29.070 ± 0.023 l	1.060 ± 0.006 i
	10	9.955 ± 0.014 c	42.105 ± 0.026 bc	1.465 ± 0.009 c	36.480 ± 0.064 f	1.725 ± 0.003 a
	15	10.140 ± 0.017 b	45.805 ± 0.020 a	1.655 ± 0.014 b	43.575 ± 0.014 b	1.821 ± 0.001 a
	20	11.100 ± 0.046 a	47.080 ± 0.023 a	1.855 ± 0.014 a	54.080 ± 0.040 a	1.971 ± 0.012 a
Carbon dots (CDs)	1	8.465 ± 0.009 j	43.185 ± 0.020 b	1.145 ± 0.003 i	25.285 ± 0.032 p	0.975 ± 0.003 k
	5	8.135 ± 0.009 n	40.610 ± 0.035 cde	0.955 ± 0.014 m	26.565 ± 0.043 o	1.260 ± 0.006 d
	10	8.265 ± 0.014 l	35.510 ± 0.040 h	1.055 ± 0.003 k	29.155 ± 0.020 l	1.350 ± 0.012 c
	15	8.400 ± 0.006 k	38.465 ± 0.020 fg	1.185 ± 0.003 h	33.175 ± 0.026 j	1.460 ± 0.006 b
	20	9.090 ± 0.012 g	39.665 ± 0.020 efg	1.230 ± 0.006 g	35.095 ± 0.049 h	1.240 ± 0.006 de
Essential oil (EO)	1	8.465 ± 0.009 j	43.185 ± 0.020 b	1.145 ± 0.003 i	25.285 ± 0.032 p	0.975 ± 0.003 k
	5	8.285 ± 0.009 l	42.420 ± 0.029 bc	1.085 ± 0.003 j	27.900 ± 0.012 m	1.060 ± 0.006 i
	10	8.455 ± 0.014 j	39.635 ± 0.020 efg	1.205 ± 0.009 h	35.205 ± 0.020 g	1.155 ± 0.003 g
	15	8.935 ± 0.020 h	40.060 ± 0.017 def	1.300 ± 0.006 f	38.870 ± 0.017 e	1.195 ± 0.003 f
	20	9.520 ± 0.017 d	47.485 ± 2.861 a	1.395 ± 0.009 d	43.215 ± 0.020 c	1.035 ± 0.003 j
Nanoencapsulated EO (EONCs)	1	8.465 ± 0.009 j	43.185 ± 0.020 b	1.145 ± 0.003 i	25.285 ± 0.032 p	0.975 ± 0.003 k
	5	8.190 ± 0.006 m	42.045 ± 0.038 bcd	1.040 ± 0.006 kl	27.135 ± 0.009 n	1.120 ± 0.000 h
	10	8.280 ± 0.012 l	37.920 ± 0.012 g	1.125 ± 0.003 i	31.210 ± 0.017 k	1.170 ± 0.006 g
	15	8.815 ± 0.014 i	39.640 ± 0.023 efg	1.240 ± 0.006 g	34.535 ± 0.038 i	1.220 ± 0.023 e
	20	9.465 ± 0.014 e	41.195 ± 0.020 bcde	1.365 ± 0.003 e	40.145 ± 0.026 d	1.060 ± 0.006 i

Values are presented as mean ± SE (*n* = 3). Within each column, means followed by different lowercase letters are significantly different according to Duncan's multiple range test (*p* < 0.05).

#### 3.16.2 Phenylalanine ammonia-lyase

A similar response pattern was observed for PAL activity (Table 3). PAL remained relatively stable during the initial stages of storage but increased markedly in untreated arils during prolonged refrigeration, reaching 47.08 U g^−1^ FW at day 20. Free EO-treated samples exhibited a comparable late-stage increase in PAL activity (47.49 U g^−1^ FW), whereas CDs- and EONCs-treated fruit maintained significantly lower PAL activities throughout storage. Differences among treatments became increasingly pronounced during the latter storage period, coinciding with the onset of accelerated senescence and oxidative deterioration in untreated samples.

#### 3.16.3 Peroxidase and polyphenol oxidase

Peroxidase and polyphenol oxidase activities exhibited pronounced storage-dependent increases, particularly in untreated arils (Table 3). PPO activity in control samples increased markedly from 25.29 U g^−1^ FW at day 1 to 54.08 U g^−1^ FW at day 20, representing the highest activity among all treatments. A comparable trend was observed for POD, suggesting progressive activation of oxidative metabolism during storage. In contrast, CDs- and EONCs-treated arils maintained significantly lower PPO and POD activities throughout the storage period, with the greatest differences becoming apparent at days 15 and 20. Free EO treatment exhibited intermediate enzyme activities but consistently remained lower than those of untreated samples at corresponding storage intervals.

#### 3.16.4 Superoxide dismutase

SOD activity was initially similar among treatments (0.975 U g^−1^ FW) but subsequently showed distinct storage-dependent patterns (Table 3). In control arils, activity increased to 1.060, 1.725, 1.821, and 1.971 U g^−1^ FW on days 5, 10, 15, and 20, respectively. In CD-treated arils, SOD activity increased to 1.260 on day 5, peaked at 1.460 U g^−1^ FW on day 15, and declined to 1.240 U g^−1^ FW on day 20. The EO treatment produced a smaller increase, reaching 1.195 U g^−1^ FW on day 15 before decreasing to 1.035 U g^−1^ FW, whereas EONC-treated arils reached 1.220 U g^−1^ FW on day 15 and declined to 1.060 U g^−1^ FW by day 20. Thus, the control exhibited the greatest late-storage SOD activity, while EO and EONCs maintained the lowest activities on day 20.

### 3.17 Microbial load

Microbial quality was significantly influenced by the interaction between packaging treatment and storage duration (Table 4). Total mesophilic bacterial populations increased progressively throughout refrigerated storage in all treatments; however, the magnitude of proliferation differed markedly among packaging systems. Control samples exhibited the most pronounced microbial growth, increasing from below detectable levels at day 1 to 5.25 log CFU g^−1^ by day 20. In contrast, CDs-treated arils demonstrated the strongest antibacterial efficacy, restricting bacterial populations to 2.85 log CFU g^−1^ at the end of storage. EONCs and free EO treatments also significantly inhibited bacterial proliferation relative to the control, although their antimicrobial effectiveness was comparatively lower than that of CDs during the advanced stages of storage.

**Table 4:** Interaction effect of treatment and storage time on microbial load and membrane stability.

Treatment	Day	Mesophilic bacteria (log CFU g^−1^)	Yeast & mold (log CFU g^−1^)	Ascorbic acid (mg 100 g^−1^ FW)	Electrolyte leakage (%)
Control	1	0.50 ± 0.00 e	0.50 ± 0.00 e	10.58 ± 0.25 a	21.55 ± 0.15 j
	5	2.15 ± 0.03 c	3.35 ± 0.08 c	10.00 ± 0.01 b	26.10 ± 0.06 h
	10	3.30 ± 0.01 b	4.55 ± 0.03 b	9.48 ± 0.38 c	29.35 ± 0.03 f
	15	3.95 ± 0.03 b	7.75 ± 0.05 a	8.72 ± 0.97 e	35.25 ± 0.08 c
	20	5.25 ± 0.08 a	9.55 ± 0.03 a	7.51 ± 0.14 g	39.10 ± 0.12 a
Carbon dots (CDs)	1	0.50 ± 0.00 e	0.50 ± 0.00 e	10.58 ± 0.02 a	21.55 ± 0.84 j
	5	0.50 ± 0.00 e	0.50 ± 0.00 e	10.05 ± 0.11 b	24.15 ± 0.08 i
	10	1.20 ± 0.06 d	2.45 ± 0.08 d	9.79 ± 0.33 c	26.55 ± 1.06 h
	15	1.65 ± 0.05 d	3.15 ± 0.03 c	9.46 ± 0.47 d	29.60 ± 0.30 f
	20	2.85 ± 0.009 c	4.85 ± 0.07 b	8.92 ± 0.01 f	31.25 ± 0.25 e
Essential oil (EO)	1	0.50 ± 0.00 e	0.50 ± 0.058 e	10.58 ± 0.36 a	21.55 ± 0.15 j
	5	0.50 ± 0.001 e	0.50 ± 0.01 e	10.47 ± 0.96 a	22.25 ± 1.09 j
	10	1.75 ± 0.05 d	3.65 ± 0.05 c	10.36 ± 0.87 a	24.00 ± 0.10 i
	15	2.75 ± 0.05 c	4.15 ± 0.06 b	9.78 ± 0.13 c	28.15 ± 0.36 g
	20	4.35 ± 0.05 b	6.85 ± 0.02 a	9.24 ± 0.01 d	33.70 ± 0.20 d
Nanoencapsulated EO (EONCs)	1	0.50 ± 0.00 e	0.50 ± 0.01 e	10.58 ± 0.10 a	21.55 ± 0.78 j
	5	0.50 ± 0.01 e	0.50 ± 0.00 e	10.30 ± 0.50 a	22.90 ± 0.05 j
	10	1.80 ± 0.05 d	3.30 ± 0.08 c	10.17 ± 0.96 b	26.00 ± 0.08 h
	15	2.10 ± 0.06 c	3.55 ± 0.03 c	9.92 ± 0.01 c	27.15 ± 0.69 g
	20	3.65 ± 0.05 b	5.65 ± 0.01 b	9.45 ± 0.06 d	30.20 ± 0.10 f

Values are presented as mean ± SE (*n* = 3). Within each column, means followed by different lowercase letters are significantly different according to Duncan's multiple range test (*p* < 0.05).

A similar trend was observed for yeast and mold populations. Control samples reached 9.55 log CFU g^−1^ after 20 days of storage, indicating extensive fungal colonization, whereas CDs effectively suppressed fungal growth to 4.85 log CFU g^−1^, followed by EONCs (5.65 log CFU g^−1^). Significant differences among treatments became evident from day 10 onward, suggesting that nano-enabled packaging systems provided sustained antimicrobial protection during prolonged refrigerated storage.

### 3.18 Ascorbic acid content

Ascorbic acid content decreased progressively during refrigerated storage in all treatments, although the extent of degradation differed significantly among packaging systems (Table 4). Control samples exhibited the most pronounced decline, reaching the lowest concentration of 7.51 mg 100 g^−1^ FW at day 20. In contrast, nano-enabled treatments effectively mitigated vitamin C depletion, with EONCs- and EO-treated arils retaining significantly higher ascorbic acid levels (9.45 and 9.24 mg 100 g^−1^ FW, respectively) at the end of storage. CDs-treated fruit also maintained higher ascorbic acid concentrations than untreated samples throughout the storage period.

### 3.19 Electrolyte leakage

Electrolyte leakage increased progressively during refrigerated storage in all treatments, indicating a gradual loss of membrane integrity associated with senescence and oxidative deterioration (Table 4). Control arils exhibited the highest EL values throughout storage, reaching 39.10% at day 20, whereas significantly lower leakage levels were observed in CDs- (31.25%), EO- (33.70%), and EONCs-treated samples (30.20%). Differences among treatments became statistically significant from day 10 onward, suggesting that nano-enabled packaging systems effectively mitigated membrane disruption during prolonged refrigerated storage.

### 3.20 Chemical composition of Persian hogweed EO

The chemical composition of Persian hogweed EO was determined by GC–MS analysis, and compounds were identified based on retention time (RT) and relative peak area percentages. A total of 30 compounds were detected, representing nearly 100% of the total chromatographic area (Table 5 and Fig. 3). The EO was dominated by aliphatic esters, which constituted the major chemical class. The most abundant compound was hexyl butanoate (30.60%), eluting at an apex retention time (RT) of 10.63 min. The second major constituent was n-octyl acetate (25.99%; RT 11.14 min). Together, these two compounds accounted for more than 56% of the total oil composition, indicating a strong ester-dominant profile. Another prominent compound was ethylidene cyclohexane (7.70%; RT 10.73 min), followed by butanoic acid, octyl ester (7.27%; RT 15.76 min). Moderate concentrations were observed for butanoic acid, 2-methyl-, octyl ester (3.36%) and butanoic acid, 2-methyl-, hexyl ester (2.27%). Minor yet notable aromatic constituents included *E*-anethole (2.07%) and p-cymene (1.30%).

**Table 5:** Relative percentage composition (% area) and retention characteristics of compounds detected in *Heracleum persicum* (Persian hogweed) essential oil.

Compound	Apex RT	Start RT	End RT	Area (%)
sopropyl 2-methylbutanoate	3.5	3.46	3.58	1.66
1-methylethyl 3-methylbutanoate	3.62	3.58	3.73	2.12
Isobutyl butanoate	3.95	3.92	4	0.15
Isovaleric acid, butyl ester	4.64	4.56	4.67	0.3
Butyric acid, isobutyl ester	4.72	4.7	4.8	0.28
3-Methyl,2-butenoic acid,isopropyl ester	4.93	4.89	5.08	0.81
n-Butyl n-butyrate	5.56	5.52	5.64	0.89
Octanol	5.83	5.79	5.9	1.03
1-Hexyl acetate	5.93	5.9	6.04	0.74
p-Cymene	6.34	6.3	6.51	1.3
Butyl 2-methylbutyrate	6.57	6.53	6.67	0.48
n-Butyl isovalerate	6.73	6.68	6.82	0.42
γ-Terpinene	7.09	7.04	7.22	1.41
Linalool	8.29	8.22	8.56	1.4
n-Hexyl isobutyrate	9.27	9.22	9.41	1.36
Hexyl butanoate	10.63	10.43	10.67	30.6
Ethylidenecyclohexane	10.73	10.67	10.94	7.7
n-Octyl acetate	11.14	10.98	11.24	25.99
Butanoic acid, 2-methyl-, Hexyl ester	11.67	11.61	11.78	2.27
Hexyl isovalerate	11.84	11.8	11.92	0.35
*E*-Anethol	13.43	13.39	13.63	2.07
n-Octyl isobutyrate	14.53	14.46	14.64	1.54
Amylvinylcarbinyl butyrate	15.4	15.33	15.51	1.3
Hexyl hexoate	15.66	15.56	15.67	1.38
Butanoic acid, octyl ester	15.76	15.68	15.87	7.27
Butanoic acid, 2-methyl-, octyl ester	16.82	16.74	16.93	3.36
n-Octyl 2-Methyl Butyrate	16.98	16.93	17.06	0.45
Hexanoic acid, 9-decen-1-yl ester	20.51	20.43	20.61	1.05
Octyl octanoate	24.89	24.82	24.97	0.32

**Fig. 3:**
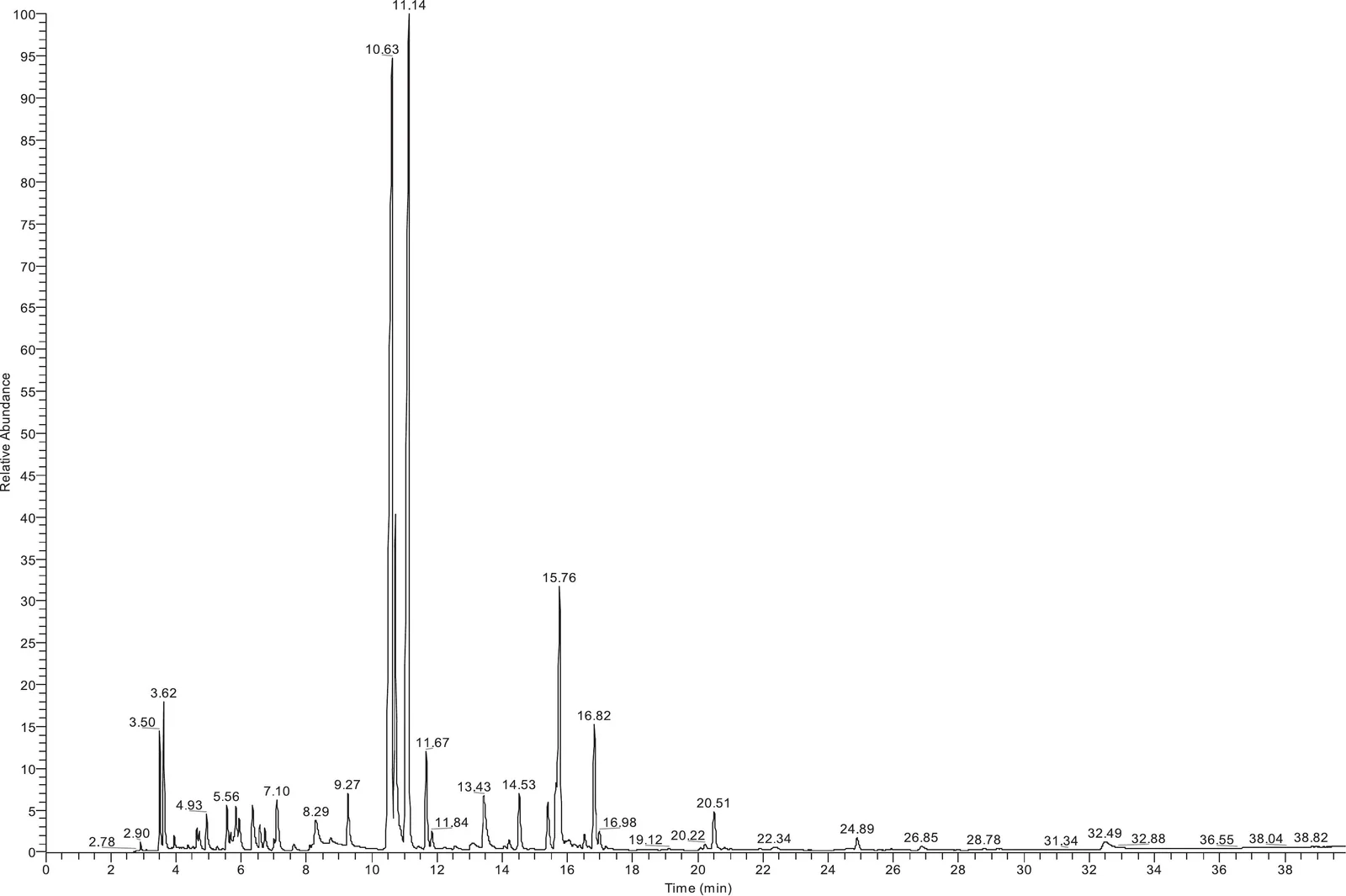
GC–MS analysis of *Heracleum persicum* (Persian hogweed) essential oil, illustrating the separation and relative abundance of volatile constituents.

Monoterpene hydrocarbons such as γ-terpinene (1.41%) and oxygenated monoterpenes such as linalool (1.40%) were detected in relatively low concentrations compared to ester compounds. Alcohols such as octanol (1.03%) and various short-chain esters including isopropyl 2-methylbutanoate (1.66%), 1-methylethyl 3-methylbutanoate (2.12%), and amylvinylcarbinyl butyrate (1.30%) were also present. Long-chain fatty acid esters such as hexyl hexanoate (1.38%), n-octyl isobutyrate (1.54%), and hexanoic acid, 9-decen-1-yl ester (1.05%) were detected in minor amounts. Trace components (<1%) included isobutyl butanoate (0.15%), isovaleric acid butyl ester (0.30%), hexyl isovalerate (0.35%), octyl octanoate (0.32%), and others (Table 5).

### 3.21 In vitro release kinetics of EO

The release profiles differed markedly between EO-loaded and nanoencapsulated- EO-loaded electrospun microfibers (Fig. 4). The non-encapsulated EO exhibited rapid initial release, reaching 27% after 1 h, 58% after 6 h, and 85% after 24 h. Release subsequently approached completion, reaching 93% at 48 h and 100% at 120 h. In contrast, the nanoencapsulated EO showed a slower and more sustained release pattern. Only 7%, 21%, and 46% of the encapsulated EO was released after 1, 6, and 24 h, respectively. Its cumulative release gradually increased to 62% at 48 h, 74% at 72 h, and 90% at 120 h. These profiles indicate that nanoencapsulation reduced the initial burst release and prolonged EO availability within the electrospun microfibers.

**Fig. 4:**
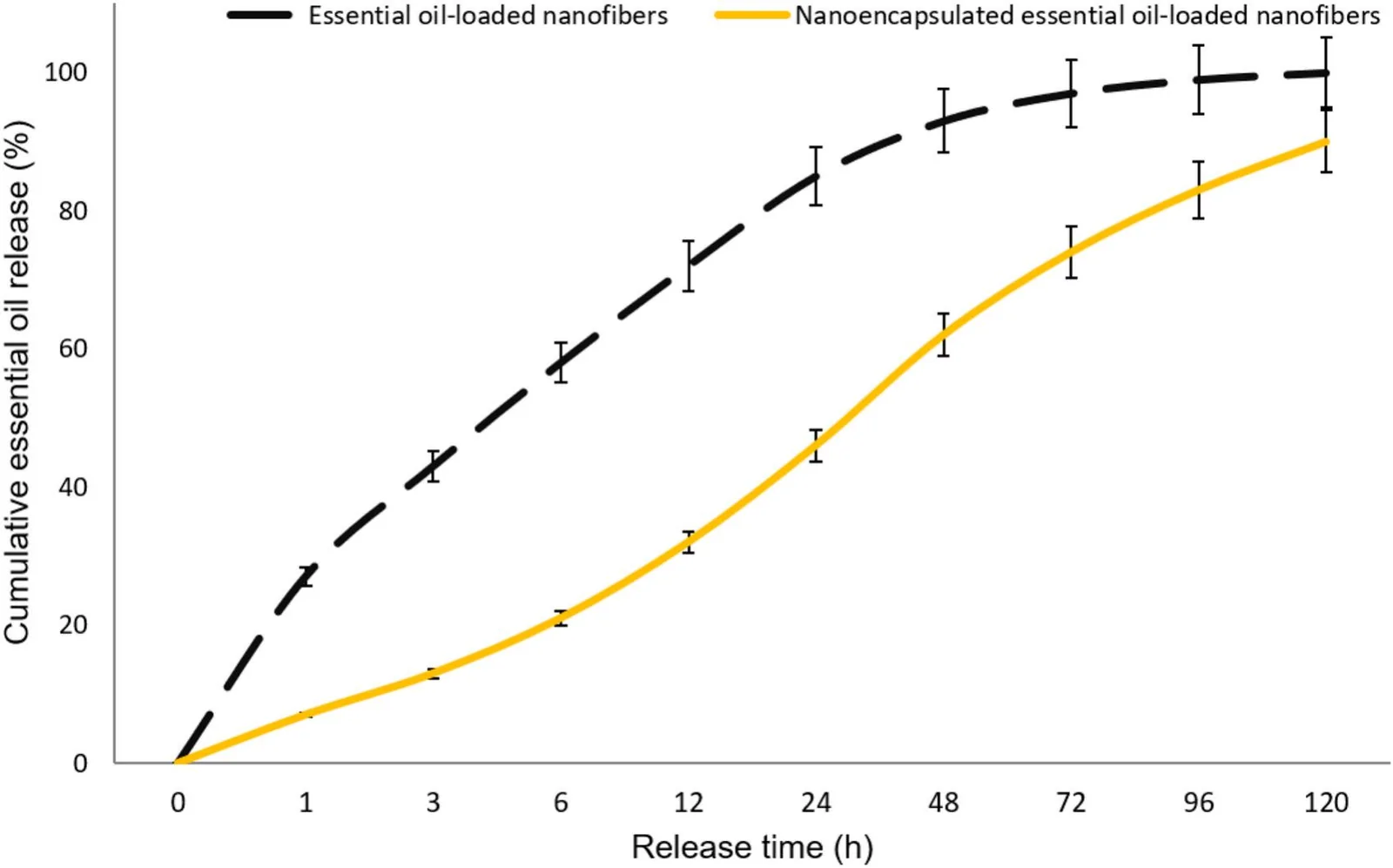
Cumulative release profiles of Persian hogweed essential oil from essential-oil-loaded and nanoencapsulated-essential-oil-loaded poly(vinyl alcohol)/chitosan electrospun microfibers over 120 h. Error bars represent standard deviations of three replicates.

### 3.22 Correlation analysis

Pearson correlation analysis revealed clear relationships among the physicochemical, biochemical, enzymatic, and microbial attributes of pomegranate arils (Fig. 5). After Benjamini-Hochberg false-discovery-rate correction, weight loss was strongly and positively correlated with electrolyte leakage (*r* = 0.96), yeast and mold count (*r* = 0.96), polyphenol oxidase activity (*r* = 0.95), and mesophilic bacterial count (*r* = 0.93). Conversely, weight loss was negatively correlated with ascorbic acid (*r* = −0.94), total phenolic content (*r* = −0.94), antioxidant capacity (*r* = −0.93), total soluble solids (*r* = −0.88), and firmness (*r* = −0.86).

**Fig. 5:**
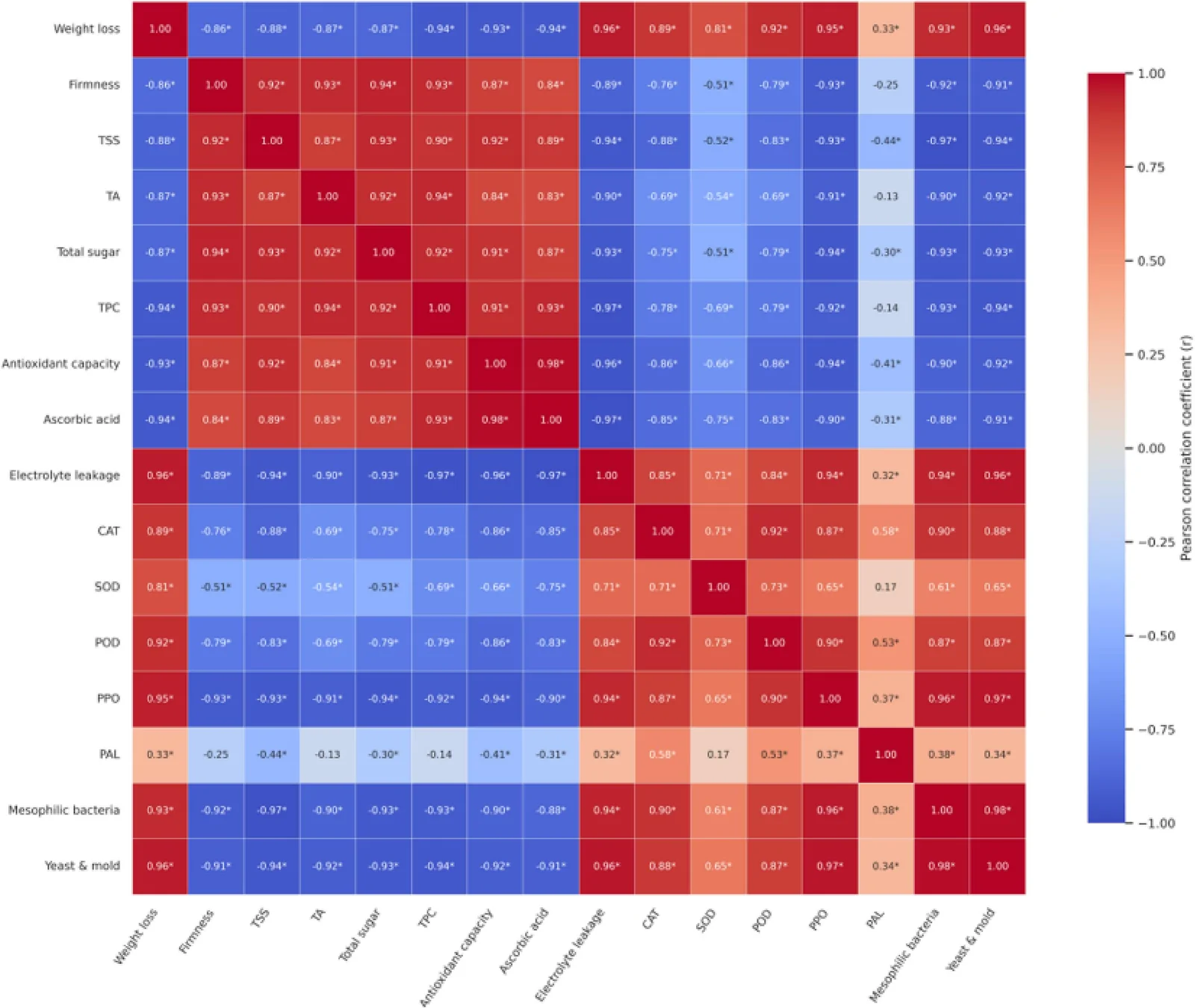
Pearson correlation heatmap showing relationships among the physicochemical, biochemical, enzymatic, and microbial attributes of fresh-cut pomegranate arils across treatments and storage times. Values represent Pearson correlation coefficients calculated from 60 pooled observations. Red and blue indicate positive and negative correlations, respectively. Asterisks denote correlations significant after Benjamini–Hochberg false-discovery-rate correction (q < 0.05). (For interpretation of the references to color in this figure legend, the reader is referred to the web version of this article.)

Electrolyte leakage was positively associated with yeast and mold count (*r* = 0.96), mesophilic bacteria (*r* = 0.94), and polyphenol oxidase activity (*r* = 0.94). It showed strong negative correlations with ascorbic acid (*r* = −0.97), total phenolic content (*r* = −0.97), antioxidant capacity (*r* = −0.96), and total soluble solids (*r* = −0.94). Antioxidant capacity was strongly correlated with ascorbic acid (*r* = 0.98), while mesophilic bacterial and yeast and mold counts were also closely related (*r* = 0.98). Firmness was positively correlated with total sugar (*r* = 0.94), titratable acidity (*r* = 0.93), total phenolic content (*r* = 0.93), and total soluble solids (*r* = 0.92).

Among the enzymatic variables, catalase activity was strongly associated with peroxidase activity (*r* = 0.92), while polyphenol oxidase activity was positively correlated with yeast and mold (*r* = 0.97) and mesophilic bacteria (*r* = 0.96). Phenylalanine ammonia-lyase generally exhibited weaker relationships with the other attributes. Collectively, these correlations demonstrate that increasing membrane deterioration and microbial proliferation were accompanied by losses in firmness, antioxidant compounds, and physicochemical quality. Because the analysis pooled treatment and storage-time observations, the correlations describe coordinated changes and should not be interpreted as evidence of causality.

## 4 Discussion

The structural and optical characterization results confirmed that the CDs possess abundant oxygen-containing functional groups, including hydroxyl, carbonyl, and ether moieties, which impart strong surface hydrophilicity and promote effective dispersion within aqueous and polymeric matrices. FTIR analysis indicated the presence of reactive carboxyl and hydroxyl groups capable of interacting with chitosan and PVA chains, thereby enhancing compatibility within the microfiber system (Jalvandi et al., 2017). XRD analysis revealed a predominantly amorphous or turbostratic carbon structure with short-range graphitic ordering, characteristic of CDs and favorable for the formation of surface defect states (Li, Qin, et al., 2024). Photoluminescence spectroscopy demonstrated a strong excitation at 388 nm and green emission at 502 nm with a large Stokes shift, indicating stable fluorescence behavior associated with oxygen-related surface defects. Collectively, these physicochemical and optical features suggest that the CDs function as redox-active nanomaterials capable of modulating reactive oxygen species, contributing to antimicrobial activity, and supporting the enhanced preservation performance observed when incorporated into electrospun microfiber packaging systems.

The uniform morphology of the control PVA/chitosan microfibers confirms appropriate polymer blending and electrospinning stability (Salleh et al., 2025). The absence of bead formation suggests optimal viscosity and electrostatic balance. Incorporation of CDs slightly increased surface roughness without disrupting fiber continuity, indicating good compatibility between nanocarriers and the polymer network. This compatibility likely results from hydrogen bonding interactions between CD surface functional groups and chitosan/PVA chains. In contrast, incorporation of pure EO induced bead formation and fiber irregularity, which can be attributed to reduced solution viscosity and increased volatility (Stoleru & Brebu, 2021). EOs often act as plasticizers, disrupting electrospinning jet stability. However, nanoencapsulation of the EO restored fiber homogeneity and reduced bead formation. This indicates improved dispersion and controlled release potential. The improved morphology of nanoencapsulated systems is particularly important, as fiber uniformity directly influences mechanical strength, moisture barrier properties, and release kinetics. Therefore, structural stabilization through nanoencapsulation likely contributed to the enhanced storage performance observed in treated samples (Li, Qin, et al., 2024).

The progressive weight loss observed during storage represents a combined effect of transpiration-driven moisture diffusion and respiration-associated metabolic activity (Yu et al., 2025). However, the significantly lower mass loss in CD- and EONCs-treated fruit indicates that nano-enabled systems modified the internal package microenvironment in a manner that limited water vapor transmission and respiration intensity (Tian et al., 2025). The electrospun microfiber pads likely acted as semi-permeable matrices capable of regulating humidity gradients while simultaneously exerting antimicrobial and antioxidant functions.

This moisture retention effect is mechanistically linked to membrane stability and oxidative control. Reduced electrolyte leakage in treated fruit indicates preservation of membrane phospholipid integrity, which directly minimizes uncontrolled water efflux (Shah et al., 2025). Therefore, weight loss reduction is not solely a barrier phenomenon but also a biochemical consequence of improved membrane stability and reduced lipid peroxidation.

The observed increase in pH and decline in titratable acidity in Control samples further reflects accelerated metabolic turnover of organic acids during senescence. Organic acids serve as respiratory substrates; thus, their depletion corresponds with enhanced respiration rates and microbial proliferation (Chidi et al., 2018). Nano-treated fruit maintained significantly lower pH increases and higher acidity retention, suggesting reduced metabolic consumption of organic acids. This stabilization of acidity is mechanistically connected to microbial inhibition. Organic acid preservation limits microbial growth, while reduced microbial proliferation decreases metabolic byproducts that elevate pH (Theron & Lues, 2007). Thus, physicochemical stability and microbial suppression operate in a feedback loop that delays senescence progression.

Color preservation provides another integrative indicator of oxidative balance. The significant retention of L*, chroma, and controlled shifts in a* and b* in nano-treated fruit directly correlate with reduced PPO and POD activity. Enzymatic browning requires substrate availability (phenolics), oxygen, and enzyme activation. By reducing oxidative stress and microbial-triggered enzyme induction, nano-treatments preserved pigment integrity (Demircan & Velioglu, 2024). Importantly, membrane integrity (lower electrolyte leakage) restricts compartmental mixing of phenolics and PPO, further limiting browning reactions. Therefore, color stability results from the combined effects of oxidative modulation, membrane preservation, and enzyme suppression (Zhang et al., 2025).

The decline in TSS and total sugars observed in Control fruit reflects intensified carbohydrate metabolism and respiration-driven depletion of soluble substrates during storage (Dladla & Workneh, 2025). Sugars function as primary respiratory fuels; therefore, their reduction is directly associated with organic acid consumption and increased oxidative metabolism. As storage progressed, untreated fruit exhibited accelerated metabolic turnover, leading to pronounced sugar loss (Brizzolara et al., 2020). In contrast, the significantly slower decline in CD- and EONCs-treated fruit indicates that nano-enabled systems effectively moderated respiratory intensity and delayed substrate utilization.

This metabolic moderation can be attributed to interconnected mechanisms operating simultaneously within treated fruit. First, suppression of microbial proliferation reduced exogenous respiratory activity, thereby limiting sugar utilization by contaminating microorganisms (Zaman et al., 2025). Second, decreased oxidative stress in nano-treated samples reduced the energetic demand for stress-related repair processes. Under high oxidative pressure, cells divert carbohydrates toward maintenance metabolism and antioxidant regeneration; therefore, the mitigation of ROS accumulation in treated fruit likely contributed to preservation of soluble solids (Meitha et al., 2020). The late-stage increases in total carbohydrates and protein probably reflect concentration caused by water loss, increased extraction of soluble carbohydrates, and accumulation of stress-related proteins rather than net biosynthesis. Similar storage-related changes have been reported in citrus and pomegranate tissues (Lin et al., 2024). Therefore, these increases should be interpreted cautiously and confirmed using dry-weight-based measurements.

CDs, owing to their redox-active surface functional groups, may function as electron mediators capable of stabilizing intracellular redox homeostasis (Kapoor et al., 2015). By reducing excessive reactive oxygen species formation, CDs indirectly limit respiration-driven sugar depletion (Rowley, 2014). Meanwhile, the controlled release of Persian hogweed EO components from nanoencapsulated systems likely exerted sustained antimicrobial effects, reducing microbial-driven metabolic drain. The combination of oxidative modulation and microbial inhibition thus provides a coherent explanation for improved retention of TSS and sugars in treated fruit.

Total phenolic content and antioxidant capacity exhibited strongly parallel trends throughout storage, reinforcing their biochemical interdependence. In Control fruit, the sharp decline in phenolics coincided with elevated PPO and PAL activities. PPO catalyzes oxidation of phenolic substrates, leading to their depletion and browning development, while increased PAL activity reflects stress-induced activation of the phenylpropanoid pathway (Kumar et al., 2023). However, under prolonged oxidative stress, phenolic degradation may exceed biosynthetic replenishment, resulting in net phenolic loss.

Nano-treated fruit maintained significantly higher phenolic levels and antioxidant capacity, indicating reduced oxidative degradation (Haydar et al., 2023). The sustained antioxidant capacity observed particularly in EONCs-treated fruit suggests that the gradual release of ester-rich EO compounds provided continuous radical scavenging support. This exogenous antioxidant contribution likely complemented endogenous defense systems, preserving phenolic substrates and limiting enzymatic oxidation.

The pronounced induction of CAT, SOD, PAL, POD, and PPO activities in Control fruit during the later stages of storage reflects a classical oxidative stress response associated with postharvest senescence (Wang et al., 2024). SOD and CAT constitute the primary enzymatic defense against ROS, converting superoxide radicals into hydrogen peroxide and subsequently into water and oxygen. Their elevated activities in untreated fruit indicate excessive ROS generation and disruption of cellular redox homeostasis (Wang et al., 2018). Importantly, such heightened antioxidant enzyme activity should not be interpreted as improved physiological performance; rather, it represents a compensatory response to intensified oxidative pressure and cellular damage.

In contrast, nano-treated fruit exhibited significantly moderated enzyme induction throughout storage, suggesting that oxidative stress levels were inherently lower. CDs, enriched with oxygen-containing functional groups such as hydroxyl and carbonyl moieties, may act as redox-active nanomaterials capable of scavenging free radicals or facilitating electron transfer reactions (Mansuriya & Altintas, 2021). By attenuating ROS accumulation at an early stage, CDs likely reduced the requirement for enzymatic detoxification. Similarly, nanoencapsulated EO treatments may have contributed indirectly by suppressing microbial proliferation, thereby limiting microbial-induced oxidative stress and metabolic burden.

Microbial proliferation is widely recognized as a major driver of postharvest deterioration, particularly in minimally processed fruit. The substantial increase in mesophilic bacteria and yeast-mold populations observed in Control fruit provides a clear explanation for the accelerated biochemical and physiological decline recorded during storage (Ahmed et al., 2023). Among the treatments, CDs exhibited the strongest antimicrobial effect, significantly suppressing both bacterial and fungal growth throughout storage. Several mechanisms may explain this activity. First, the nanoscale size and high surface area of CDs facilitate close interaction with microbial cell membranes, potentially causing structural disruption (Anand et al., 2019). Second, surface oxygen-containing functional groups may participate in redox reactions that generate localized oxidative stress in microbial cells (Chen et al., 2026). Third, electrostatic interactions between charged CD surfaces and negatively charged microbial membranes may destabilize membrane integrity (Li, Ma, et al., 2024).

EO treatments also reduced microbial populations; however, this activity should be interpreted in relation to the ester-dominant composition of Persian hogweed EO rather than mechanisms typically assigned to phenolic monoterpenes. The dominant aliphatic esters are electrically neutral and sufficiently lipophilic to partition into the hydrophobic region of microbial membranes. Their alkyl chains promote affinity for membrane lipids, whereas the ester carbonyl can interact with the more polar interfacial region. Accumulation within the bilayer may disturb lipid packing and membrane fluidity, increase nonspecific permeability, promote leakage of ions and low-molecular-weight metabolites, and impair membrane-dependent respiration and energy generation. Microbial esterases may additionally hydrolyze some esters into their corresponding alcohols and organic acids, which could intensify membrane and intracellular acidification stress; nevertheless, this pathway remains mechanistically plausible rather than directly demonstrated in the present study. Previous investigations have confirmed antimicrobial activity in ester-rich *H. persicum* oils dominated by compounds such as hexyl butyrate, octyl acetate, and 2-ethylhexyl acetate, although their potency can be lower than that of phenolic-rich EOs (Ghavam, 2023; Mustafavi et al., 2022). The antioxidant effect should also be interpreted cautiously because aliphatic esters lack the readily donated phenolic hydrogen required for strong hydrogen-atom-transfer radical scavenging. Consequently, the preservation of antioxidant activity in treated arils probably resulted from the combined action of minor oxygenated constituents, alcohols and unsaturated compounds, synergistic interactions within the complete EO, and indirect suppression of oxidative stress rather than strong radical scavenging by the major esters alone. Nanoencapsulation further increased the effectiveness of this ester-rich oil by reducing volatilization and oxidative loss, improving its dispersion within the microfibrous matrix, and sustaining the release of active constituents during storage (Yousefi et al., 2024). Prolonged microbial inhibition reduced microbial respiration and consumption of sugars and organic acids, thereby supporting the retention of soluble solids, total sugars, and acidity. It also limited microbially induced membrane degradation and reactive oxygen species formation, which explains the lower electrolyte leakage, moderated antioxidant-enzyme responses, and improved preservation of phenolic compounds and tissue integrity in nano-treated arils.

The significantly higher retention of ascorbic acid in nano-treated fruit therefore provides strong biochemical evidence of improved redox homeostasis. In untreated samples, accelerated ROS production likely promoted ascorbate oxidation, leading to its rapid depletion (Vacca et al., 2004). In contrast, the elevated ascorbic acid levels observed particularly in EONCs- and EO-treated fruit indicate that oxidative pressure was effectively mitigated. This protective effect may result from a synergistic interaction between endogenous antioxidant systems and exogenously supplied antioxidant compounds from Persian hogweed EO. Controlled release of ester-rich and minor phenolic constituents may have provided continuous radical scavenging support, thereby preserving the ascorbate pool and stabilizing cellular redox balance over extended storage (Basavegowda & Baek, 2021).

Electrolyte leakage measurements provide complementary structural confirmation of this biochemical stabilization. Increased leakage in Control fruit reflects disruption of membrane phospholipids and loss of semipermeable barrier function, typically resulting from lipid peroxidation under oxidative stress (Xing et al., 2022). In nano-treated fruit, significantly lower leakage values indicate preservation of membrane integrity and reduced oxidative damage to lipid bilayers (Parente et al., 2026). Meanwhile, membrane integrity is intrinsically linked to water retention and overall physicochemical stability. Intact membranes regulate transmembrane water movement and minimize uncontrolled moisture efflux (Martínez-Ballesta & Carvajal, 2016). Consequently, the lower electrolyte leakage observed in nano-treated fruit is consistent with reduced weight loss and moderated dehydration dynamics. This structural stabilization not only preserves tissue firmness but also limits concentration effects that can distort biochemical measurements during storage.

The different responses to CDs and EONCs may reflect their distinct modes and durations of activity. The stronger microbial suppression by CDs could be associated with their small size, high surface area, and surface functional groups, which may promote rapid contact with microbial cells and disrupt membrane integrity; however, because the experiment was conducted in darkness and direct microbial-membrane or ROS measurements were not performed, this interpretation remains tentative (Khan et al., 2024). In contrast, the superior preservation of phenolics, antioxidant capacity, and vitamin C by EONCs may result from protection of volatile EO constituents against evaporation and degradation, followed by their gradual release during storage. *H. persicum* EO-loaded chitosan nanoparticles have previously shown biphasic release and prolonged antioxidant activity (Yousefi et al., 2023), while EO nanocarriers have preserved phenolics and ascorbic acid in refrigerated fruit (Vakili-Ghartavol et al., 2024). Thus, CDs may have provided a stronger immediate antimicrobial effect, whereas EONCs offered longer-lasting protection of oxidation-sensitive compounds; nevertheless, confirmation requires direct measurements of CD–microbial interactions, EO release, nanoparticle migration, and oxidative-damage markers.

## 5 Conclusion

The present study demonstrates that nano-enabled electrospun packaging systems incorporating carbon dots (CDs) and nanoencapsulated Persian hogweed essential oil (EONCs) constitute an effective multifunctional strategy for preserving the postharvest quality of fresh-cut pomegranate arils during refrigerated storage. Structural analyses confirmed that the CDs possessed abundant oxygen-containing surface functionalities, pronounced photoluminescent properties, and a predominantly amorphous carbonaceous framework, supporting their redox-regulating potential. Moreover, incorporation of CDs and EONCs into PVA/chitosan electrospun microfibers preserved fibrous morphology and enhanced structural homogeneity relative to free EO-loaded systems.

Nano-enabled treatments significantly mitigated quality deterioration by reducing electrolyte leakage and moisture loss, preserving titratable acidity, maintaining color attributes, and slowing the depletion of soluble solids and sugars. In parallel, treated arils exhibited superior retention of total phenolics, antioxidant capacity, and ascorbic acid, particularly under EONCs and CDs treatments. The moderated activities of CAT, SOD, POD, PPO, and PAL further indicated that nano-enabled packaging alleviated oxidative burden and maintained cellular redox homeostasis, thereby minimizing the need for compensatory activation of antioxidant and phenylpropanoid metabolism. Additionally, substantial inhibition of mesophilic bacteria, yeasts, and molds confirmed the pronounced antimicrobial efficacy of the developed packaging systems, with CDs exhibiting the strongest inhibitory effect.

The preservation mechanisms involved attenuation of reactive oxygen species accumulation, sustained release of EO bioactives, maintenance of membrane integrity, suppression of microbial proliferation, and stabilization of primary and secondary metabolic processes. These complementary mechanisms effectively delayed senescence and quality deterioration, particularly during prolonged storage. The integration of redox-active carbon nanomaterials and controlled-release EO nanocarriers within electrospun biopolymeric matrices therefore represents a promising next-generation active packaging platform for enhancing the nutritional, physicochemical, and microbiological stability of minimally processed fruits. Future studies should focus on release kinetics, toxicological assessment, consumer acceptance, and commercial-scale implementation to facilitate industrial translation of this technology.

## Availability of data and material

All data generated or analyzed during this study will be made available by the corresponding author upon reasonable request.

## CRediT authorship contribution statement

**Sahar Ashari:** Writing – original draft, Methodology, Formal analysis. **Raheleh Ebrahimi:** Writing – review & editing, Supervision, Formal analysis. **Weria Weisany:** Writing – review & editing, Visualization, Supervision, Project administration, Methodology, Data curation. **Shima Yousefi:** Writing – review & editing. **Orang Khademi:** Writing – review & editing.

## Consent to participate

Not applicable.

## Consent for publication

Not applicable.

## Ethics approval

This study involved plant-derived materials and fresh-cut fruit and did not include human participants or experimental animals. Therefore, approval by an institutional ethics committee was not required.

## Code availability

Not applicable.

## Funding

This research received no specific grant from funding agencies in the public, commercial, or not-for-profit sectors.

## Declaration of competing interest

The authors declare that they have no known competing financial interests or personal relationships that could have influenced the work reported in this manuscript.

## Data Availability

Data will be made available on request.
